# Connectivity and population structure of albacore tuna across southeast Atlantic and southwest Indian Oceans inferred from multidisciplinary methodology

**DOI:** 10.1038/s41598-020-72369-w

**Published:** 2020-09-24

**Authors:** Natacha Nikolic, Iratxe Montes, Maxime Lalire, Alexis Puech, Nathalie Bodin, Sophie Arnaud-Haond, Sven Kerwath, Emmanuel Corse, Philippe Gaspar, Stéphanie Hollanda, Jérôme Bourjea, Wendy West, Sylvain Bonhommeau

**Affiliations:** 1 Institut Français de Recherche pour l’Exploitation de la Mer, Délégation de La Réunion, Rue Jean Bertho, BP 60, 97 822 Le Port Cedex, La Réunion France; 2 ARBRE, Agence de Recherche pour la Biodiversité à la Réunion, 34 avenue de la Grande Ourse, 97434 Saint-Gilles, La Réunion France; 3 IRD, UMR MARine Biodiversity Exploitation and Conservation (MARBEC), Saint-Clotilde, La Réunion France; 4 Department of Genetics, Physical Anthropology and Animal Physiology, University of the Basque Country (UPV/EHU), Barrio Sarriena s/n, 48940 Leioa, Spain; 5 CLS, Sustainable Management of Marine Resources, 11 rue Hermès, 31520 Ramonville Saint-Agne, France; 6 IRD, UMR MARBEC, Fishing Port, Victoria, Seychelles; 7 IFREMER, Station de Sète, Avenue Jean Monnet, CS 30171, 34203 Sète Cedex, France; 8 Department of Environmental Affairs, Private Bag X2, Vlaeberg, 8018 South Africa; 9 Department of Biological Sciences, University of Cape Town, Rondebosch, Cape Town, 7701 South Africa; 10 Mercator Ocean, 10 rue Hermès, 31520 Ramonville Saint-Agne, France; 11 Seychelles Fishing Authority (SFA), Fishing Port, Victoria, Seychelles; 12 Present Address: UMR INRAE-UPPA 1224 Ecobiop, Aquapole, 173 RB918 Route de Saint-Jean-de-Luz, 64310 Saint-Pée-sur-Nivelle, France; 13 Sustainable Ocean Seychelles (SOS), BeauBelle, Mahe, Seychelles; 14 Centre Universitaire de Formation et de Recherche de Mayotte, MARBEC, 8 rue de l′Université BP53, 97660 Dembeni, Mayotte

**Keywords:** Computational models, Ecology, Population genetics

## Abstract

Albacore tuna (*Thunnus alalunga*) is an important target of tuna fisheries in the Atlantic and Indian Oceans. The commercial catch of albacore is the highest globally among all temperate tuna species, contributing around 6% in weight to global tuna catches over the last decade. The accurate assessment and management of this heavily exploited resource requires a robust understanding of the species’ biology and of the pattern of connectivity among oceanic regions, yet Indian Ocean albacore population dynamics remain poorly understood and its level of connectivity with the Atlantic Ocean population is uncertain. We analysed morphometrics and genetics of albacore (n = 1,874) in the southwest Indian (SWIO) and southeast Atlantic (SEAO) Oceans to investigate the connectivity and population structure. Furthermore, we examined the species’ dispersal potential by modelling particle drift through major oceanographic features. Males appear larger than females, except in South African waters, yet the length–weight relationship only showed significant male–female difference in one region (east of Madagascar and Reunion waters). The present study produced a genetic differentiation between the southeast Atlantic and southwest Indian Oceans, supporting their demographic independence. The particle drift models suggested dispersal potential of early life stages from SWIO to SEAO and adult or sub-adult migration from SEAO to SWIO.

## Introduction

Albacore tuna (*Thunnus alalunga,* Scombridae) is an important, commercially harvested pelagic species with high migratory capacity, distributed throughout most tropical and temperate oceans, except in the polar regions^1^. The commercial albacore catch is the highest globally among all temperate tuna species, contributing around 6% in weight to global tuna catches over the last decade^2,3^ and 5% in 2017^4^. Although currently not subject to overfishing in the South Atlantic and Indian Oceans^5,6^, there is significant uncertainty about stock assessments due to the lack of key biological information^7,8^.

The Indian and the South Atlantic Oceans remain the two least known areas regarding albacore population structure and connectivity. Life history, biology and population structure information are critical input for stock assessments and the representation of these parameters has to resemble the actual population structure of the resource^9^. In this context ‘population’ is used in the biological and ecological sense (individuals of a species that live simultaneously in a geographical area and have the ability to reproduce). Regional Fisheries Management Organizations (RFMOs) currently manage albacore based on six hypothetical populations or stocks (Mediterranean, North Atlantic, South Atlantic, Indian, North Pacific, and South Pacific). The definition of these populations or stocks have been supported by some genetic studies^10–15^ but the definition remains controversial, particularly within an ocean (e.g. between albacore populations of northern and southern hemispheres). Additionally, differences in delimitation among various methods (i.e. serological, parasitological, proteomic, tagging, morphometric, genetic) is evident (see Table 1 and 2 in Nikolic and Bourjea^16^).

Tag-recapture experiments suggest low rates of migration between hemispheres^13^. The albacore remains underrepresented in ongoing programs such as the Atlantic Ocean Tuna Tagging Program AOTTP, as these target mostly tropical species such as bigeye, skipjack and yellowfin tuna. Consequently, migratory exchange between the South Atlantic and Indian Oceans remains unresolved.

The establishment of an accurate population boundary requires a multidisciplinary approach to which population genetics can provide an important contribution^17,18^. In fact, genetic markers are widely used to investigate connectivity between populations and to define stocks and the degree of mixture between stocks in a fishery^19–21^. Based on catch statistics, Morita^22^ suggested active inter-oceanic migration of adult albacore between Atlantic and Indian Oceans off South Africa, which could be promoted by the strong Agulhas Current, as also suggested for the congeneric bigeye tuna (*Thunus obesus*)^23^. Albacore from southwest Africa are usually genetically clustered with the Atlantic population^15,22^. Also, in a previous genetic study^24^ based on samples of albacore from the seas off the Cape of Good Hope (South Africa), southeast Atlantic and southwest Atlantic observed no heterogeneity. Yet, to our knowledge, no samples from the southwest Indian and southeast Atlantic Oceans were analyzed to test for the existence of inter-oceanic migration. The scarce information on the population structure of albacore in the South Atlantic and Indian Oceans thus warrants further analyses of the population structure and connectivity of this species in these areas, in order to test Morita’s hypothesis of a significant exchange.

Genetics studies on albacore began with assessing the population structure of this species in the Pacific and Atlantic Oceans^9–12,15,24–28^; and more recently, in the Indian Ocean using multi-locus analyses based on nuclear and mitochondrial genetic markers. These multi-locus analyses include microsatellite markers, which have been increasingly used during the last decade in fisheries management^29^. Although recent studies on albacore have used Single Nucleotide Polymorphism (SNPs, e.g.. 616 and 75 SNPs^30,31^), the limited number of SNPs used for this species has not provided high-density genetic map coverage and genome information .

In addition to genetic markers, fish populations can also be discriminated based on variations of morphometric traits^32^, such as length–weight relationships, which can be the result of either genetic variation and/or phenotypic responses to variations in local environmental factors^33–35^. Few length–weight relationship estimates are available for albacore in the Indian Ocean^36^.

Dispersal (and thus connectivity) is greatly dependent upon ocean fronts and currents^37^. Since the population structure of marine pelagic fishes is influenced by physical features of the marine environment, biophysical modelling can be used to predict passive dispersal^38^. Lagrangian transport models can help to predict the potential early stage dispersal pattern and extent of connectivity from spawning area to settlement sites^39,40^, including over large scales^41^, and to assess oceanographic model limitations^42^. Here, we used Lagrangian simulation of particles solely drifting with surface currents (i.e. with no active locomotion) to test if connectivity through passive drift can explain, at least partly, early life stage dispersal of albacore tuna and the genetic structure of the population.

The objective of our study was to investigate the connectivity and population structure of albacore tuna between the southeast Atlantic (SEAO) and southwest Indian Oceans (SWIO), with multiple methods. We collected genetic and morphometric data from albacore in 2013 and 2014 in four geographic regions to determine genetic or morphometric differences. We combined these information with the Lagrangian simulation to test if connectivity through passive drift can explain, at least partly, early life stage dispersal of albacore tuna and the genetic structure of the population.

## Materials and methods

### Sampling

**Figure 1:**
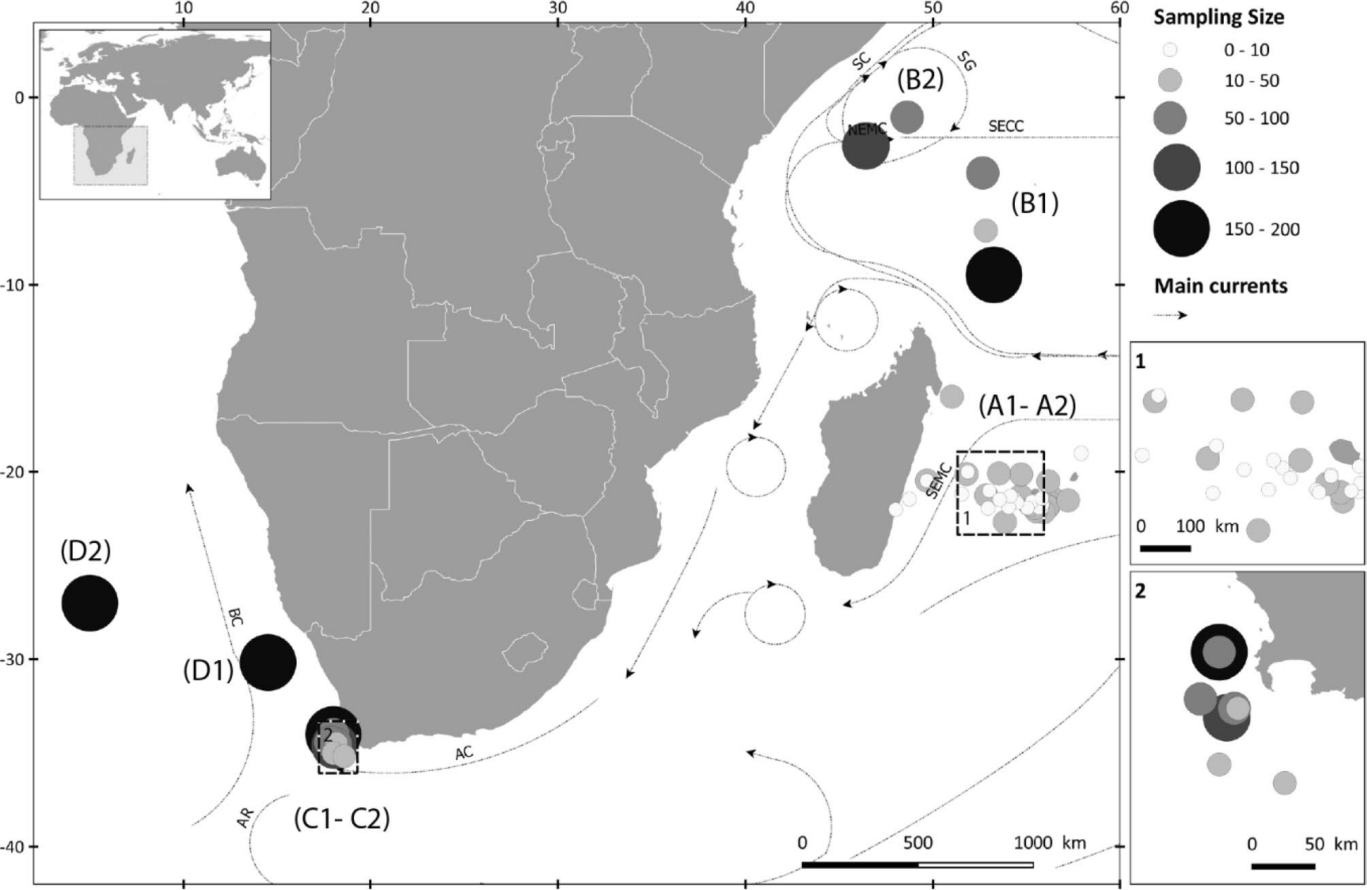
Sampling locations of albacore sampled for genetic analysis (total of 1,874 individuals). Circles are proportional to the number of individuals collected in both periods. Austral summer (1) and Austral winter (2). (A) East Madagascar (around Reunion Island (A1) n = 236; around Reunion Island and east of Madagascar (A2) n = 230). (B) North Madagascar ((B1) n = 233; (B2) n = 233). (C) South Africa ((C1) n = 323; (C2) n = 276). (D) Southeast Atlantic Ocean ((D1) n = 157; (D2) n = 191). Southwest Indian Ocean (SWIO) also mentioned by (A) and (B) sampling locations. Southeast Atlantic Ocean (SEAO) also mentioned by (C) and (D) sampling locations. Benguela Current (BC), Agulhas Current (AC), Agulhas Return Current (AR), Somali Current (SC), Southern Gyre (SG), South Equatorial Counter Current (SECC), and Southeast Madagascar Current (SEMC).

Albacore samples were collected from four different geographic regions from June 2013 to August 2014 over two seasons (periods); in the southwest Indian Ocean between the east of Madagascar and Reunion (region A), and Seychelles to the coast of Somalia (region B), in South African waters (region C), and in the southeast Atlantic Ocean (region D) (Fig. 1). To account for the variability of environmental conditions and life history traits, sampling was performed over two seasons (Austral summer: November-February, i.e. potential reproduction season of tunas; and austral winter: April–August, i.e. potential feeding period or post-reproduction period^8,43^) except in geographic region B, and over two years (2013 and 2014) except in geographic region D (see Appendix 1). They are called sampling locations (A1, A2, B1, B2, C1, C2, D1, and D2) (Fig. 1). Fish from the waters of geographic region A were sampled on a research trip on board a commercial longliner and from artisanal fishermen using vertical longlines in the second season. Albacore from the geographic region B were caught by purse-seiners and sampled during processing. Finally, in geographic regions C and D, the samples were obtained from the catch landed by the commercial pole-and-line fishing boats and at sea by observers. No ethical approval was required as all fish sampled were dead by sampling time. A total of 1,874 adults’ individuals were collected for genetic analyses, 2,129 with body length, and 1,059 with weight information (Appendix 1). The geographic positions were also collected per fishing operation for geographic regions A, B, and C, and trip for geographic region D.

Based on the sampling locations and morphometric analysis, three scenario clusters were proposed to analyse data: a scenario T1, where each area would harbour distinct stocks (regions A, B, C, and D), a scenario T2, where individuals caught in the same area during potential reproductive and feeding seasons would belong to distinct stocks (regions A-B and C-D), and a scenario T3, where individuals caught on either side of the Cape of Good Hope (South Africa) during the potential reproductive and feeding seasons would belong to different groups (regions A1, A2, B1, B2, C1, C2, D1, and D2). Both morphometric and genetic analyses were designed to identify the most likely among those three scenarios.

### Morphometric analysis

Macroscopic analyses of the gonads and stomach contents were carried out within the GERMON project (see report^43^). These confirmed that the areas studied in the southwest Indian Ocean (regions A and B) are indeed breeding and feeding areas for the species (see Appendix 1). No gonad samples were available from region D and the individuals of region C were mainly immature. The proportion of females to males, and matures to immatures (< 90 cm FL), based on the reproduction study of albacore in the southwest Indian Ocean^44^ and previous studies^45–47^, were mapped per region and season using ArcGIS software (https://www.arcgis.com) (Appendix 2).

Fork length (FL, cm) and weight (W, kg) were measured per individual, then data were tested for normality with Kolmogorov–Smirnov normality tests with the Lilliefors correction^48^, and for homogeneity of variances with Levene tests^49^. Sizes and weights were compared among geographic regions and sexes with nonparametric k-sample permutation tests^50^. The analysis was carried out using the R statistical computing version 3.3.0. and included the following libraries (“perm”^51^; “car”^52^).

A common problem in fisheries research is to decide if the parameters from a simple linear regression fit are different between populations^53^. Analysis of length–weight relationships per sampling case T1 were performed using the R statistical software with FSA^53^ and MASS^54^ packages. The exponential equation W = *a* * FL^*b*^ was fitted to determine the relationship between FL and W, where, *a* is the coefficient related to body form and *b* is the exponential expressing relationship between length–weight, also called allometric coefficient^55,56^. Parameters were estimated using a Nonlinear Least Squares (NLS) method from the function of Rossiter^57^ to provide more precise relationships than the classical relations. The coefficient of determination (R^2^) was used as an index of the goodness of fit of the estimates and standard error was calculated for parameter estimations. Graphical analysis was performed for the comparison between geographic regions with reported allometric equations.

To determine if the parameter estimates are statistically different among geographic regions, sexes and seasons, the length–weight model was transformed to a linear model by taking the natural logarithms, log(Wt) = log(*a*) + *b*log(FLt) + *ϵ*t, with y = log(W), x = log(FL), slope = *b*, and intercept = log(*a*). We then determined whether log(*a*) and/or *b* differs mainly between geographic regions and sexes using analysis of covariance (ANCOVA). A test of whether the fish in a population exhibit isometric growth or not can be obtained by noting that *b* is the estimated slope from fitting the transformed length–weight model^53^. The following statistical hypotheses (H0: b = 3 “Isometric growth”; ⇒ H1: b ≠ 3 “Allometric growth”) were tested using t-tests from the linear regression results.

### Molecular analysis

#### Technical protocols

The genomic DNA was isolated from a tissue sample of muscle (25 ng) without fat and skin using Qiagen DNeasy spin columns and quantified with NanoDrop (Thermo Fisher Scientific).

Microsatellite PCRs were performed on selected loci (see description of the selection process in supplementary text S1-a, S2-b, and Appendix 3) in 25 μl reactions containing 5 ng of template DNA, 1X reaction buffer, 1.5 mM MgCl2, 0.24 mM dNTP, 0.1 μM of each primer, and 1U Taq polymerase. The PCR cycling for microsatellite markers consisted of an initial denaturation at 95 °C for 10 min, followed by 40 cycles: denaturation at 95 °C for 30 s, annealing at the appropriate temperature (55 °C) for 30 s, and extension at 72 °C for 1 min and a final extension at 72 °C for 10 min. Each PCR had a negative control as well as a positive control. The PCR products were genotyped with Applied Biosystems 3,730 XL and the profiles obtained were analysed using GeneMapper^®^ v5.0 software. Allele binning was performed using the bins created in the study by Nikolic et al.^58^ and adding alleles respecting the allele size (± 0.4 bp). The corresponding type of repetition (e.g. di and tri) was respected as much as possible.

#### Population genetic diversity and differentiation

For the purpose of this paper we define “migration” as the movement of individuals from one place to another, “dispersal” as the process or result of the spreading of individuals from one place to another and “gene flow” as the transfer of genetic variation from one population to another. We use the term “gene flow” when *Nm* (number of efficient migrants entering a population by generation) is estimated and “migration rate” when *m* (fraction of individuals sampled in a given group that are more likely migrants derived from another population of origin) is estimated.

##### Nuclear data

Genetic analyses were performed using individuals clustered a priori according to each of the putative stock scenarios (T1-3) (number of alleles, heterozygosity, and *F*IS; similar to previously described analyses per marker see text S1-a). We added analysis of *F*IS by bootstrapping (1,000) to obtain 95% confidence intervals (CI) with GENETIX. Hardy Weinberg Equilibrium (HWE) tests in each population and in the overall data were carried out using GENEPOP v4.0 software^59^ with Markov chain parameters (10,000 dememorization, 1,000 batches, and 10,000 iterations per batch) (hypothesis Ho = random union of gametes) with an alternative hypothesis (H1 = heterozygote deficit).The U Score test was used as it is more powerful than the probability-test especially when there is inbreeding or population admixture^60^. Q-test analysis are preferable to maintain a proper balance between the avoidance of type I errors and the induction of type II errors^61–65^, so we added this analysis using Q-value R package as it performs the false discovery rate (FDR)^61–65^. We completed our understanding on deviation of HWE by using HWxtest V.1.1.9 R package^66^ because in most tests for Hardy–Weinberg proportions (with real data and multiple alleles) there are often rare alleles which make the asymptotic test unreliable. By using the genotype counts contained within each test, we extracted and re-analysed the data to plot the distribution with Monte Carlo sample size, for a smoother curve.

Genetic differentiation among samples within each scenario (T1-3) was estimated as pairwise Wright’s F-statistics (*F*ST)^67^ using ARLEQUIN 3.1^68^ computed with 10,000 permutations and significance level at 0.05. *F*ST average with 95% confidence interval (CI) and standard deviation (SD) was computed using GENETIX^69^ with 1,000 bootstrap. Representation of genetic matrix distance (*F*ST) and Principal Component Analysis on allelic frequencies was performed using the R package ADEGENET^70,71^. Isolation by distance (IBD) in sampling scenario T2 was tested using a Mantel test between genetic (*F*ST and Euclidean Edwards’ distance) and geographic distances with 10,000 resampling between individuals and geographic regions using ADEGENET, ADE4^72^ and GRDEVICES (R Development Core Team and contributors worldwide) R packages. Finally, IBD differentiation (patches) was performed using a 2-dimensional kernel density estimation with MASS R package.

The hierarchical genetic structure and the admixture rates were estimated by the Bayesian individual clustering assignment performed with StRUCTURE 2.3.4 software^73^ with the admixture model and correlated allele frequencies. The number of clusters, *k*, was determined by comparing log-likelihood ratios in 5 runs for values of *k* between 1 and 14 (number of main geographic sampling + 1) with a burn-in period of 100,000 steps followed by 1,000,000 MCMC replicates. For obtaining optimal *k*, results were analysed through StructureSelector^74^ (see https://lmme.ac.cn/StructureSelector/) using the methods of^73,75,76^, and^77^.

Bifurcated evolutionary trees were built using POPTREEW^78^ with 100,000 bootstrapping samples and *D*SW genetic distances. GenGIS 2^79^ was used to build a 3D phylogeography tree with sample geographic site information and phylogenetic tree from POPTREE2^80^ file results. Additional analysis (AMOVA, network analysis etc., see supplementary text S1-b) were performed in order to check for consistency of the results when based on different a priori.

##### Test for sex-bias

We used a population assignment test for sex-biased dispersal using the software GenAlEx v. 6^81^. This method produces Assignment Index correction (AIc) values for each sex according to Mossman and Waser^82^. Mean negative AIc values characterize individuals with a higher probability of being immigrants, whereas positive values characterize individuals with a lower probability of being migrants. The statistical analysis was carried out using the R statistical computing version 3.3.0^83^ and included the following libraries (“lawstat”^84^; “ade4”^85^; “mgcv”^86,87^; and “glmmADMB”^88,89^). AIc values for each sex were compared with a non-parametric Mann–Whitney U-test (also known as the Mann–Whitney–Wilcoxon, MWW) because samples violated assumptions of the normality and homoscedasticity (homogeneity of variance) (Shapiro’s, Levene's and Brown and Forsythe's tests).

##### Combining molecular and morphologic analysis

A fork length measurement was available for all but five individuals genotyped in this work. The length–weight relationship estimated previously (Appendices 4, 5), was used to estimate the lengths of the five fish without a length measurement. We used the assignPOP R package^90^ to perform population assignment using a machine-learning framework and employed genetic and non-genetic (morphometric) data sets, evaluating the discriminatory power of data collected. We tested the power of assignment with 1 to 8 hypothetical populations. We used principle component analysis (PCA) for dimensionality reduction; Monte-Carlo cross-validation to estimate mean and variance of assignment accuracy; K-fold cross-validation to estimate membership probability; resample individuals and loci either randomly and based on locus FST value; machine-learning classification algorithms, including LDA (Linear Discriminant Analysis) and SVM (Support Vector Machine). The output was visualized using ggplot2 functions.

The package assignPOP was also used to standardize the data with and without the optional software to remove low variance loci across the dataset. The default setting of variance threshold is 0.95, meaning that a locus will be removed from the dataset if its major allele occurs in over 95% of individuals across the populations. A low variance locus—which has a major allele in most individuals and a minor allele in very few individuals—is not likely to be useful because an allele that only occurs in the training or test data will not help ascertain population membership of test individuals.

### Particle-tracking simulation design

#### Passive drift simulations

Larvae, juvenile and young albacore (less than 80–90 cm LF) are thought to be less able to perform vertical migration as their swim bladder is not yet functional^91^ and young albacore predominantly inhabit shallow water (< 50 m)^92^, hence simulations of surface water movements are relevant to track dispersal. Moreover, spawning takes place at the sea-surface ^93,94^. To compute the trajectories of passively drifting individuals, we used the modelled surface current fields from the GLORYS-1 (G1) reanalysis of the World Ocean circulation^95^ performed by the Mercator-Ocean centre (https://www.mercator-ocean.fr/) with the NEMO numerical ocean model (https://www.nemo-ocean.eu/). The G1 model has a horizontal resolution of 0.25° and 50 vertical layers. The G1 reanalysis provides a close-to-reality, 3-dimensional, simulation of the World Ocean dynamics as it assimilates satellite altimetry, temperature and salinity measurements. Passive drift trajectories were computed using the Lagrangian trajectory simulation software ARIANE (freely available at https://www.univ-brest.fr/lpo/ariane/) and the G1-simulated currents in the first model layer (surface currents). One geographic region per day was recorded for analyses of trajectories. This trajectory simulation technique was previously used to study the passive dispersal of hatchlings (and then juveniles) from the west Atlantic leatherback turtle (*Dermochelys coriacea*) population^96,97^.

We chose to simulate the dispersal of particles drifting only with surface currents. Passive drift is the most parsimonious hypothesis for exploring early stages (i.e. egg, larvae (1 month cohort) and small juveniles (three monthly cohort) connectivity, given the high uncertainty about (1) the depth occupied and (2) the development of swimming ability. We performed passive drift simulations over 1 month and 3 months (young of the year individual). After the juvenile phase, fish are capable of active movement (linked to their size and habitat) in addition to being transported by oceanic currents^98^.

#### Individual release

Between 1,000 to 5,000 particles were released in each simulation but for visual reasons we presented 2000 particles in the figures. Release positions were uniformly distributed over potential spawning regions^8,36^, except in the southeast Atlantic C-D (unknown spawning region in this case, including South Africa) but it was important to test for a possible migratory pathway from SEAO to SWIO. Individuals were released daily during a 3-month period corresponding to the spawning season. Releases are performed uniformly over time (i.e. the same number of individuals was released every day). To address the interannual variability in the currents, we performed 17 simulations—one per year between 1998 (first year of available current data) and 2014 (last year of the sampling program)—for each potential spawning ground. From these simulations we then computed the average, minimal and maximal numbers of transits between the different areas.

### Ethical approval and informed consent

Fish samples authors confirm that all experiments were carried out in accordance with regulations. The field studies did not involve endangered or protected species. Albacore tuna is a commercial species all over the world that is, thus far, not subject to any ethical official rules. No specific permissions were required for the sampling locations. Fishes were sampled from French, Seychelles and South African fishing vessels (at sea with within observer program) in the authorized marine waters or at landing. Only dead fish were sampled.

## Results

### Morphometry

Length–weight data were not normally distributed across the overall data (Lilliefors test: *p* < 0.001), and the variances were heterogeneous among geographic regions (Levene test: F = 8.41, *p* < 0.001 for the size; F = 13.12, *p* < 0.001 for the weight) and sexes (Levene test: F = 19.26, *p* < 0.001 for the size; F = 9.19, *p* < 0.01 for the weight). Univariate nonparametric statistical tests revealed that sizes and weights significantly differ among regions (permutation test: chi-squared = 509.69, *p* < 0.001 for the size; chi-squared = 643.78, *p* < 0.001 for the weight) and sexes (permutation test: chi-squared = 26.76, *p* < 0.001 for the size; chi-squared = 16.3, *p* < 0.001 for the weight).

**Figure 2:**
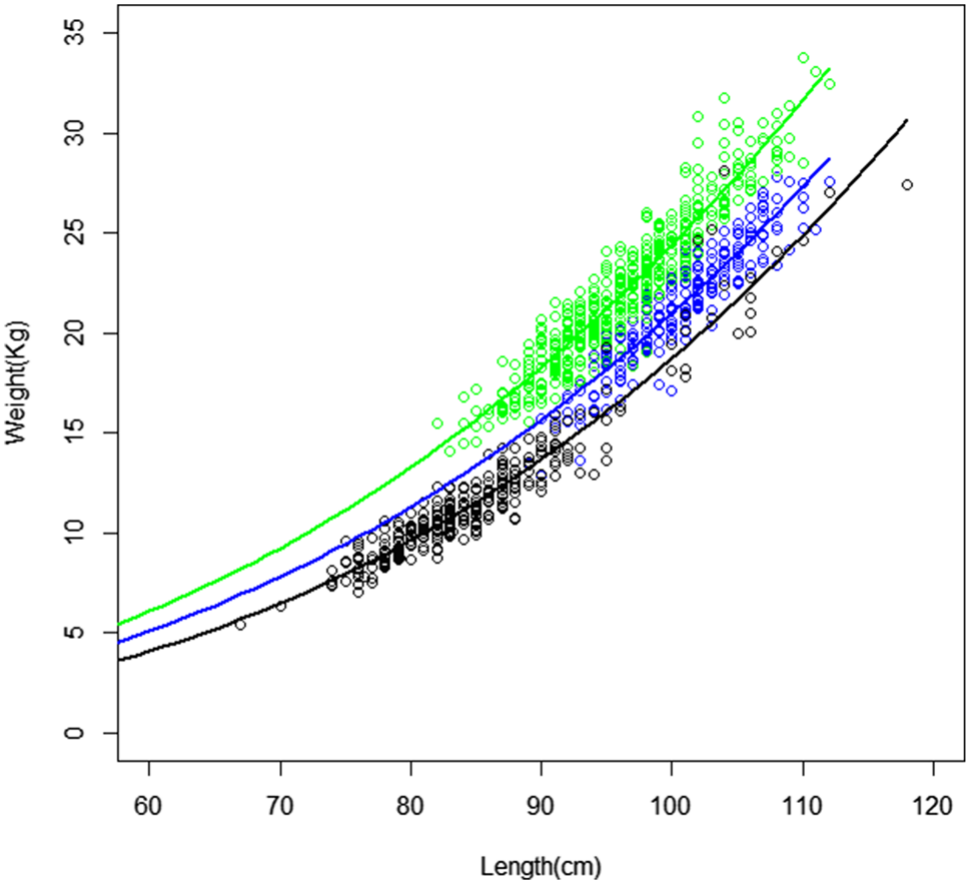
Length–weight relationship (fork length (cm) and weight (kg)) for albacore (*Thunnus alalunga)* per geographic regions from the catch data of Reunion (blue; region A), Seychelles (green; region B) and South Africa (black; region C) fishery. The curves represent the length–weight relationship according to NLS form: Seychelles (green), Reunion (blue), and South Africa (black).

Length–weight relationships in sampling scenario T1 (regions A, B, and C) revealed significant differences between geographic regions (Table 1), with a lower ratio for individuals from South Africa (Region C), probably due to the sampling of earlier life stages, and higher values for the northernmost Indian Ocean (Region B) (Fig. 2). The homogeneity test (ANCOVA) also revealed significant differences among geographic regions. Here we considered estimates of length at 50% maturity around 90 cm fork length (FL)^46^ at an age of 4–5 years. Based on length of adult fish (> 90 cm FL), individuals caught would therefore be considered as adults in geographic regions A and B, and immatures in C and D (Appendix 2). For more details on the length–weight relationship see supplementary text (S2-a).

**Table 1:** Length–weight relationships of albacore tuna according the equation weight (kg) = a*FL^*b*^ from the non-linear least squares (NLS) form per geographic regions cases.

Regions	n	a	Std. error (a)	b	Std. error (b)	R^2^	Analysis of covariance
A	269	5.9206 × 10^–5^	1.987 × 10^–5^	2.7747	7.259 × 10^–2^	0.8520	
B	485	8.4869 × 10^–5^	1.648 × 10^–5^	2.7293	4.236 × 10^–2^	0.8950	
C	302	2.0103 × 10^–5^	4.172 × 10^–6^	2.9846	4.619 × 10^–2^	0.9127	
A-B	754	5.7418 × 10^–4^	1.474 × 10^–4^	2.3007	5.583 × 10^–2^	0.6983	Intercepts significant
A-C	571	5.1527 × 10^–6^	8.293 × 10^–7^	3.2983	3.507 × 10^–2^	0.9502	Intercepts significant
C-B	787	2.6044 × 10^–6^	6.102 × 10^–7^	3.4786	5.127 × 10^–2^	0.8719	Slopes and intercepts significant
A-B-C	1,056	1.2819 × 10^–5^	2.758 × 10^–6^	3.1212	4.691 × 10^–2^	0.8390	Slopes and intercepts significant

*n* is the number of individuals, *a* the constant, *b* the allometric coefficient, R^2^ the coefficient of determination, and Std. Error the standard error. The last column summarizes the Analysis of Covariance (ANCOVA) with the linear model (General Linear Model, GLM) between geographic regions.

The results indicate differences in the length–weight relationships between geographic regions (Table 1, Fig. 2, Appendix 4). While the interaction terms are not significant between the geographic regions A and B (*p* = 0.1166), and A and C (*p* = 0.5044) (i.e. not enough evidence to conclude a difference in slopes), the *P-*value *(p)* for the indicator variable suggests that there is a difference in intercepts between these two pairs of geographic regions (*p* < 2.2 × 10^–16^). Because these geographic regions (A and B, A and C) have statistically equal slopes but different intercepts, there is a constant difference between the log-transformed weights of albacore regardless of the log-transformed lengths of albacore. Concerning geographic regions B and C, there is significant difference (in slopes and intercepts) in the length–weight relationship between the two geographic regions (*p* = 0.003334 and *p* < 2.2 × 10^–16^ respectively).

Males are significantly larger than females (Appendix 5), except in South African waters (region C), where most immature individuals were collected. The Appendix 5 summarizes the NLS-adjusted curve for each geographic region and the linear models to test sex-specific differences. The difference in length–weight relationship between the sexes is significant in geographic region A. Yet, for geographic region B, the results show differences between the sexes (Kruskal–Wallis test *p* < 0.05) that are not confirmed by the parallelism test (contained in the linear model with the length–weight relationship). Females appear heavier than males for the fork length below 99 cm. This trend reverses above 99 cm fork length. However, these differences in the length–weight relationship per sex in geographic region B are not confirmed by the analysis of variance (Appendix 5). A bias in sex ratio (proportion of females to males in the sample) in favor of females with fork length (FL) < 100 cm has been confirmed in a previous study of geographic regions A and B^36^. In geographic region C, both males and females reached smaller sizes compared to the other geographic regions, due to the dominance of juveniles in the sample.

### Molecular

#### Genetic diversity

Descriptive statistics across *loci* and samples, and for each microsatellite and sample are shown in Appendix 3. Based on the 32 microsatellite markers retained after quality selection from the initial panel of 54 putative loci (see Supplementary text S2-b), analyses were performed considering four different geographic groups (Fig. 1) under three a priori scenarios of individual grouping, T1, T2 and T3. Classic genetic variability per geographic group and scenario is described in Table 2. High expected and observed heterozygosity values were found for all samples, with values ranging from 73.7 ± 13.2 to 74.8 ± 12.8 for He and 68.3 ± 13.0 to 71.4 ± 12.7 for Ho. All samples or groupings had significant *P-*values for Hardy Weinberg tests, meaning that all scenarios resulted in a deficit of heterozygotes (Table 2). Inbreeding coefficients (*F*IS) and CI 95% were superior to 0 for all sampling locations (0.03–0.08) with the lowest values for geographic regions A and B (particularly B1), and the highest for region C (Table 2). Overall data and each population per scenario presented a significant deviation from HWE, yet with a high proportion of False Discovery Rate (> 0.45). Based on these results, we completed marker analysis using HWxtest. All markers presented a tail in the distribution and 17 markers also presented infrequent alleles creating a “shoulder” in the distribution. Those potential outcomes, in which a rare genotype occurred, can partly explain the observed deviation from HWE.

**Table 2:** Descriptive statistics for albacore samples over 32 microsatellite loci without null alleles.

Samples	n	MNa	He	Hnb	Ho	FIS
**Scenario T1**						
A	466	16.8	74.2 ± 13.0	74.3 ± 13.0	71.1 ± 12.4	**0.043 (0.033–0.052)**
B	466	16.8	73.9 ± 12.9	74.0 ± 12.9	71.4 ± 12.7	**0.035 (0.026–0.043)**
C	598	17.9	74.1 ± 13.5	74.2 ± 13.6	68.8 ± 12.4	**0.072 (0.061–0.082)**
D	344	16.9	74.8 ± 12.5	74.9 ± 12.5	70.3 ± 12.3	**0.062 (0.050–0.071)**
**Scenario T2**						
A1	236	15.4	74.3 ± 12.8	74.5 ± 12.9	71.4 ± 12.2	**0.042 (0.027–0.052)**
A2	230	14.8	73.9 ± 13.2	74.1 ± 13.3	70.8 ± 13.0	**0.045 (0.029–0.057)**
B1	233	15.4	73.7 ± 13.2	73.9 ± 13.2	71.4 ± 13.5	**0.033 (0.019–0.043)**
B2	233	14.9	73.9 ± 12.8	74.1 ± 12.8	71.3 ± 12.4	**0.038 (0.023–0.046)**
C1	322	16.0	74.1 ± 13.5	74.2 ± 13.5	69.3 ± 12.4	**0.067 (0.052–0.077)**
C2	276	15.6	73.9 ± 13.6	74.1 ± 13.7	68.3 ± 13.0	**0.079 (0.060–0.092)**
D1	156	14.8	74.8 ± 12.8	75.1 ± 12.8	70.7 ± 13.2	**0.059 (0.039–0.071)**
D2	188	14.9	74.5 ± 12.4	74.7 ± 12.4	69.9 ± 12.1	**0.064 (0.046–0.075)**
**Scenario T3**						
A-B	932	18.5	74.1 ± 13.0	74.2 ± 13.0	71.2 ± 12.5	**0.039 (0.033–0.045)**
C-D	942	19.1	74.4 ± 13.1	74.5 ± 13.1	69.4 ± 12.2	**0.068 (0.060–0.075)**

Number of genotyped individuals (n); mean number of alleles (MNa); mean percent of expected (He), expected unbiased (Hnb), and observed (Ho) heterozygosity; and inbreeding coefficient (*F*IS) with CI 95%. Significant values are in bold. Mean values are ± SE. Sample abbreviations as in Fig. 1. Different samples considering scenarios of clustering T1, T2, and T3 (sampling scenario).

POWSIM^99^ results for the 32 *loci* dataset indicated that the probability of detecting population structure was high and statistically significant at *F*ST ≥ 0.001 for χ2 and Fisher's tests. When *F*ST was set to zero (i.e. no divergence among samples), the proportion of false significant values (α type I error) was lower than the intended value of 4% for χ2 test and was 7% for Fisher's test. Markers shared a similar range of mutation rates (*u*) of around 2 × 10^–4^ (mean) and 1 × 10^–4^ (median).

#### Population structure

The clustering analysis, carried out with STRUCTURE (Appendix 6), over 5 runs, favoured the existence of two main clusters. Pairwise *F*ST values (Table 3) were significant between almost all comparisons of A/B with C/D, under all 3 scenarios, indicating differentiation between SEAO and SWIO Indian samples. Pairwise *F*ST with the scenario T2 revealed lower (and not significant) *F*ST values between eastern Madagascar (A2) and South Africa (C1), and also between Mozambique Channel (B1) and South Africa (C1) (Table 3).

**Table 3:** Pairwise *F*ST among albacore for albacore samples considering the three scenarios clustering T1, T2 and T3 over 32 microsatellite loci with 10,000 permutations.

Scenario T1	A	B	C	D				
A	**0**							
B	− 0.00005	**0**						
C	**0.00127**	**0.00143**	**0**					
D	**0.00281**	**0.00340**	0.00013	**0**				

Scenario T2	A1	A2	B1	B2	C1	C2	D1	D2
A1	**0**							
A2	0.00066	**0**						
B1	0.00044	− 0.00030	**0**					
B2	0.00022	0.00045	0.00038	**0**				
C1	**0.00141**	0.00042	0.00045	**0.00145**	**0**			
C2	**0.00272**	**0.00101**	**0.00193**	**0.00213**	− 0.00032	**0**		
D1	**0.00290**	**0.00287**	**0.00385**	**0.00310**	− 0.00100	0.00026	**0**	
D2	**0.00290**	**0.00187**	**0.00292**	**0.00278**	0.00024	− 0.00077	− 0.00145	**0**

Scenario T3	A-B	C-D						
A-B	0							
C-D	**0.00198**	0						

Significant corrected *P*-value (< 0.05) are bold.

**Figure 3:**
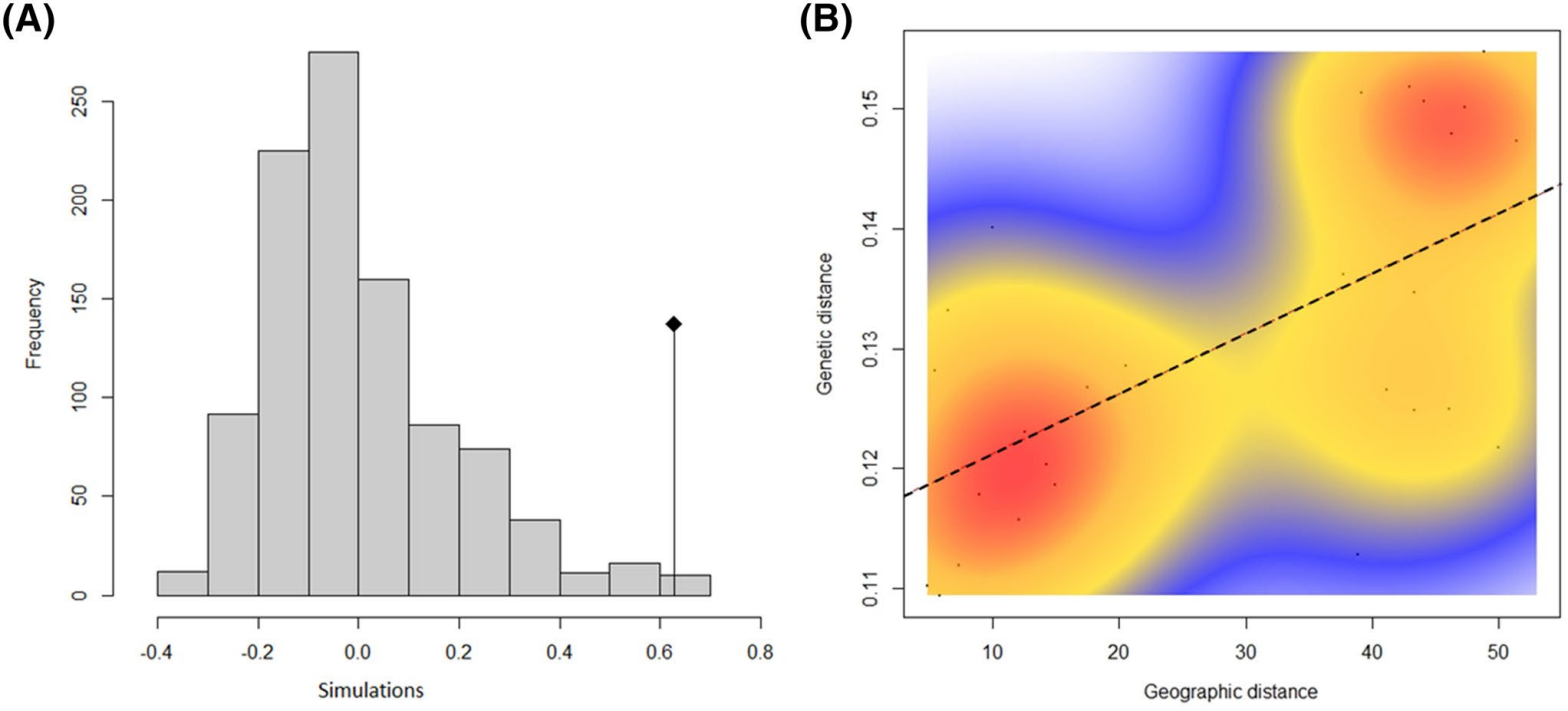
(**A**) Mantel test correlation (the original value of the correlation between the distance matrices is represented by the dot, while histograms represent permuted values (i.e., under the absence of spatial structure); here the isolation by distance is clearly significant, and (**B**) scatterplot of isolation by distance using a 2-dimensional kernel density estimation (red line is the correlation). Both analysis between Euclidian genetic and geographic distances using the sampling scenario T2 (regions A1, A2, B1, B2, C1, C2, D1, and D2).

AMOVA results revealed significant genetic differences among the two genetic clusters (A/B, and C/D) representing 20% of the total variation and among individuals within geographic area (A, B, C, and D) representing 36% of the total variation. The Mantel test confirmed a significant (*p* < 0.05) correlation between both genetic distance (*F*ST and Euclidean) and geographic distance (Fig. 3A, e.g. with Euclidean distance). However, the scatterplot showed two consistent clouds of points, thus suggesting that apparent IBD was actually mostly due to the existence of two separate entities rather than by a regular increase of genetic differentiation with geographic distances (Fig. 3B, e.g. with Euclidean distance). The 3D geophylogeny Neighbour Joining (NJ) from Dsw distances (Appendices 7 and 8) also differentiated SEAO from SWIO, in line with STRUCTURE (Appendix 6), and PCA (Appendix 9).

AssignPop analysis (Appendix 10), combining the genetics and morphometrics (length), supported the scenario of two genetic clusters k = 2 (A/B, C/D). When using the genetic-morphometric data, the assignment accuracies of regions A and B increased and that of region C remained high, resulting in increasing overall assignment accuracy (Appendix 10-B). Assignment accuracies of region B remained low (Appendix 10-B). The results were similar with removal of alleles with low variance. The addition of the variable sex did not improve the results.

#### Test for sex-biased dispersal

Analysis of sex-biased dispersal showed that males have strongly negative AIc values for groups A-B (southwest Indian Ocean), and C (South Africa), indicating that males are more often likely to be immigrants (Appendix 11). Nevertheless, the Mann–Whitney U-tests were not significant.

### Predicting connectivity through passive drift: particle-tracking modelling

We chose to present results from the simulation of the year 2009 that corresponds to a neutral phase of the Indian Ocean Dipole (IOD)^100^. IOD is an oscillation of the sea-surface temperature between the eastern and western side of the tropical Indian Ocean. Similarly to the El Nino Phenomenon in the Pacific Ocean, IOD has a strong influence on the climate-ocean system of the Indian Ocean and affects surface and subsurface currents^101^.

**Figure 4:**
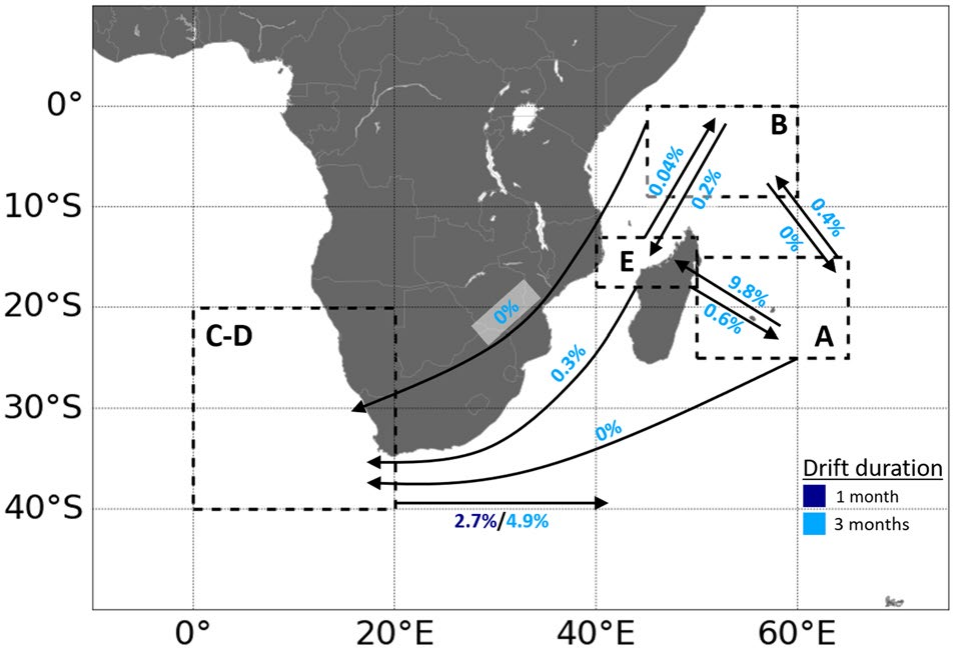
Schematic simulated passive drift trajectories for tuna larvae (and then small juveniles) released from different potential spawning areas (delineated by dotted lines): East Madagascar (A), North Madagascar (B), Southeast Atlantic (C, D), and Mozambique Channel (E).

Simulated 1- and 3 month trajectories of passive drift are shown in Appendices 12 and 13. A schematic view of the number of transits between the areas after 1 and 3 month drift is presented in Fig. 4. One month trajectories display similar spatial patterns as 3 month trajectories, but 1 month is too short to draw conclusions about the destination of the particles except for the ones released off South Africa (Appendix 12).

Modeling results suggest that connectivity through passive drift is possible, although low, between SEAO and SWIO. Passive exchange between the southeast Atlantic (C-D) and the three potential spawning grounds in the southwest Indian Ocean (A, B, E) appear to be highly asymmetric and subject to significant inter-annual variability (Fig. 4). Particles released in the SEAO can be driven very quickly (within less than 1 month) into the Indian basin by the eastward flowing Agulhas Return Current that originates from the retroflection of the Agulhas Current off the southern tip of Africa. Over the 20 individual years tested, an average proportion of 2.7% (min. = 1.7%, max. = 4.7%) of particles passed from SEAO to SWIO after 1 month of drift, and 4.9% after 3 months (min. = 2.1%, max. = 6.7%). After entering the Indian Ocean particles from the Atlantic circulate eastward around 40°S. We found no direct connectivity through early life stages between SEAO and the three potential spawning grounds of SWIO. Particles that remained in the southeast Atlantic disperse northwestward with the Benguela Current and the South Atlantic Equatorial Current. The passive dispersal route from SWIO to SEAO is longer and more complex. The Mozambique Channel appeared to be the main pathway linking the SWIO to the SEAO. Dispersal in the Mozambique Channel indicated large mesoscale activity but is mostly southward. At the southern end of this channel, water continued to move southward with the Agulhas Current. No particles entered the SEAO from the northern Mozambique Channel (E region) after 1 month of dispersal, and only a small percentage entered after 3 months (mean = 0.3%, min. = 0%, max. = 2.4%).

Passive exchanges to the SEAO were predicted to be far more limited from the East of Madagascar (A) and, surprisingly, almost non-existent from North of Madagascar (B), although located in the direct vicinity of the Mozambique Channel. In fact, most particles from B were entrained toward the Equator line and dispersed into the North Hemisphere. Within 1 month and 3 month of drift, no particles originating from areas A and B were able to reach the SEAO. The A region is strongly connected with the E region, and, to a lesser extent, with the B region, by the North Madagascar Current that flows northwestward and then goes around Cap d’Ambre, the northern tip of the island. Passive transport from A to E accounted for 9.8% (min. = 6.5%, max. = 13.4%) of particles after 3 months. In comparison, the B region was crossed in average by 0.4% of particles from A (min. = 0%, max. = 1.4%) after 3 months. Very few passive particles travelled to A region from B. Passive connectivity from B to E was very weak after 3 months of drift (mean = 0.2%, min. = 0%, max. = 0.7%). The pattern was similar in the opposite direction (from E to B), but subject to greater interannual variability: an average proportion of 0.04% (min. = 0%, max. = 0.7%) of particles transited this way after a 3 month drift. Interestingly, it appears that passive connectivity from E to B (northward drift through Mozambique Channel) was inversely proportional to the passive connectivity from E to C-D (southward drift). Drift simulations from the spawning region offshore of northwest Australia (F) indicated that passive flow from southeast to north Indian Ocean is also possible (Appendix 13). Indeed, several particles released in this geographic region were driven westward by the South Indian Equatorial Current.

## Discussion

The results presented here indicate small but significant genetic and morphometric differences between the southeast Atlantic and southwest Indian Oceans, suggesting the existence of two independent populations, and identifying a spawning ground in South Western Indian Ocean waters (eastern Madagascar). We consider these results in relation to simulations of larval dispersal through the main currents, and previous data on other species in the same geographic region, before discussing their implications under the current delineation of management units. The results have significant implications for RFMOs (e.g. ICCAT and IOTC) as the stock assessments and in turn the sustainable management of albacore relies on the accurate representation of population structure and connectivity across management units.

### Instantaneous population structure based on morphometric data

Stock identification is traditionally based on morphometric differences, sometimes combined with demographic modelling. The morphometric analyses of albacore tuna revealed differences in the weight, regardless of the length, between geographic regions from SWIO waters (regions A and B). The results indicate a greater albacore weight gain in northernmost waters, in line with previous hypotheses that the waters between the Seychelles and the coast of Somalia constitutes an important albacore feeding region^43^. Results obtained here are consistent with the enhanced phytoplankton productivity associated with the dynamic system of mesoscale eddies of the Mozambique Channel^102–104^ and mid-ocean shallow banks of the Seychelles plateau^105^, whereas the Mascarenes are characterised by low-nutrient subtropical waters^106^. Surface dwelling behaviour is reported for albacore of geographic regions B and C, whereas albacore mostly occur in deeper waters in geographic region A^43^. Others authors^107^ also found morphometric differences between southwest Indian and the southeast Atlantic albacore populations and suggested that the Agulhas current enabled the sporadic interchange of adult albacore in deep waters.

Male albacore are significantly larger than females, except in South African waters, as found in several previous studies, showing that few females of albacore tuna exceed a fork length of 100 cm^46,108^. This may be due to asymptotic growth and different natural mortality between the sexes, and/or females investing more energy into gonad development than into somatic growth. Moreover, some authors^46,109^ suggested that increased mortality would occur just after their first reproduction. Concerning the length–weight relationship, the difference between the sexes was only significant in the east of Madagascar and Reunion waters (region A). This suggests the presence of an important area of reproduction in the east of Madagascar, resulting in the higher weight of the females. This result seems consistent with large catches in the southwest Indian Ocean in the waters off eastern Madagascar^110–114^ and between 10°S and 25°S^45^, which led to assumptions of the existence of a spawning ground in the southwest Indian Ocean by Nishikawa et al.^115^, and Nishida and Tanaka^116^, also relayed by Nikolic et al.^8^. A common factor among tuna species is that spawning takes place when sea-surface temperatures^94^ reach or exceed 24 °C^93^. Spawning generally occurs throughout the year over vast areas of the Atlantic, Indian, and Pacific Oceans and in the warm northern equatorial waters, but in higher latitudes it is restricted to the summer months (^94,117,118^). Here, the adults were mainly encountered in the SWIO region, particularly east of Madagascar. These results are consistent with histological analyses of the gonads and the investigation of the gonadosomatic index^36^ corroborating that spawning occurs between 10˚S and 30˚S in the east of Madagascar from October to January.

Some authors^8^ estimated the potential spawning area in the southwest Indian Ocean basins (also positioned in western areas of subtropical gyres) by mapping the catch of longline fisheries. Albacore abundance in warm waters show a seasonal peak in November–December in the SWIO region, after which the fish would migrate to other geographic regions^8,119^. These results suggest that after the spawning season some fish migrate towards other regions.

### Long term integrated population structure based on genetic differentiation

High values of genetic diversity were observed among all sampling locations and sampling scenarios of albacore tuna. These values are close to those previously reported for both this species^11,120^ and bluefin tuna^121–123^. The positive *F*IS values obtained (0.03–0.08) are within the range of those obtained in a previous study^120^ and the confidence intervals also differed from zero. These values reflect a bias towards an excess of homozygotes that may be due to a technical pitfall resulting in partial allele amplification, or due to biological origin indicating departure from random mating. A Wahlund effect (two differentiated populations that are included in a single sample) would imply genetic structure of approximately the same level as those *F*IS values, whereas *F*ST values were much lower. The main driver of departures from HWE seem to be the presence of infrequent alleles resulting in rare genotypes, rather than genotyping error. The low differentiation value (low *F*ST), despite a clear dichotomy between SWIO and SEAO (geographic regions), is not surprising, since *F*ST is bounded by the level of diversity (here very high); its significance is thus more meaningful than the value itself. Moreover, it is the raw number of migrants (*Nm*) rather than the migration rate (*m*) that determines *F*ST, thus larger populations will tend to exhibit lower *F*ST values.

All statistical methods used here on a large dataset yielded similar results, indicating structural differentiation of two albacore genetic groups: one identified in the southwest Indian Ocean (regions A and B) and the other corresponding to southeast Atlantic Ocean (regions C and D). The genetic relationship between possible SWIO and SEAO stocks has been the subject of much debate, with conflicting results found in the literature with genetic data (e.g.^120,124^), a combination of blood markers and direct tagging^13^. Our work however agrees with and confirms, based on a large sample of the southwest Indian and southeast Atlantic albacore population, the results of different authors^30,31,124^.

Based on catch statistics, Morita^22^ suggested migration of albacore between two oceans off South Africa, which could be facilitated by the strong Agulhas Current. It is possible that the connectivity is a passive migration from the SWIO to the SEAO (through the Mozambique Channel), and an active migration in the opposite direction. These results and scenarios are consistent with oceanographic simulations on individual dispersal, yet imply a limited exchange, similar to the results of the genetic differentiation and clustering analysis.

The low but significant levels of genetic differentiation reported previously for albacore tuna have been ascribed to their population characteristics, such as reproduction in the open ocean, their highly migratory nature, and large population sizes^125,126^. Significant differentiation between samples A/B (SWIO) and C/D (SEAO), based on the analysis of allelic frequencies, suggests a very small number of migrants exchanged per generation, implying demographic independency of populations from each side of the Cape of Good Hope.

As the area of SEAO sampled by the study does not provide suitable conditions for reproduction and spawning, it might be hypothesized to support a mixed stock, with migrants from the SWIO and the Atlantic north of the Benguela System. Genetic data from elsewhere in the Atlantic during the reproduction period would be needed to test such hypothesis. In a previous study^127^, bigeye tuna from distinct stocks (Atlantic and Indo-Pacific) have been shown to be in contact around South Africa, and the distribution and mixture of fishes from each stock seems influenced by the dynamics of the currents in that area. Population genetic structuring can be influenced by ecological (e.g. homing behaviour) and physical (e.g. present-day ocean currents, past changes in sea temperature and levels) factors^23^. Repeated glaciations and deglaciations have caused changes in sea levels, temperatures^128,129^, and currents^127^ and have regularly led to changes in the distribution of marine species. Bigeye tuna showed divergent clades, which were assumed to have been originated during the last Pleistocene glacial maxima^23^. Fish population expansion during the Pleistocene has been reported in several studies^130–133^ and may lead to the modification of population structure. We thus encourage continued analyses on albacore by integrating the models of the evolutionary history worldwide.

### Predicted passive dispersal based on Lagrangian simulations

Lagrangian simulations support the possibility of passive connectivity between SWIO and SEAO regions that could shape the early dispersal of albacore populations in this part of the globe. The connectivity between the Indian and Atlantic Oceans was suggested or demonstrated in other species of tuna (e.g. Barth et al.^134^), swordfish (e.g. West^135^), sharks (e.g. Da Silva Ferrette et al.^136^), and sea turtles (e.g. green turtles^137^). One shall also note that passive simulations were unable to reproduce trajectories from SEAO to the three potential spawning regions in the SWIO region, in line with genetic data showing significant differentiation.

Offshore of the southern tip of Africa, the Agulhas Current retroflects eastward, into the Indian Ocean. The retroflection takes the form of an unstable jet that can shed warm eddies into the Atlantic Ocean. Passive trajectories from the SEAO into the SWIO rapidly reach latitudes beyond 40°S where water temperatures are relatively low (below 15 °C), well below 24–25 °C, the preferred range of albacore eggs and larvae^45,46,138,139^. These are thus unlikely to survive. Conversely, individuals carried from the SWIO into the SEAO by the warm Agulhas Current and then the Agulhas Rings would have a much better chance of survival due to higher temperatures than the ones following the opposite direction to the cold Circumpolar Current, which may explain the asymmetry of predicted passive exchange. In fact, the Mozambique Channel appears to be the only path tested where links may occur between the southwest Indian and the southeast Atlantic Oceans. Nevertheless, according to simulations over the duration of 1 to 3 months, connectivity is still predicted as very limited. Thus, the warm Agulhas current may promote passive or quasi-passive dispersal of early stage albacore from the SWIO to the SEAO regions, yet both prediction through modelling and inference through genetic data suggest this dispersal pattern, if it exists, would be very limited. In the yellowfin tuna, an asymmetric migration was supported from the Indo-Pacific to the Atlantic^134,140^, and several Indian Ocean migrants were detected at the Atlantic spawning site demonstrating asymmetric dispersal^140^, as is suggested here by modelling of albacore tuna.

Large ocean currents and physicochemical characteristics are congruent with the differentiation and connectivity between albacore of southeast Atlantic and southwest Indian Oceans. Predicted dispersal patterns reported here are indeed in line with the dichotomy observed with both morphometric and genetic data. However, no obvious parameter prevents migration of post-larval or adult albacore with partially or fully developed thermoregulation abilities from the south Atlantic to the south Indian Ocean, or the other way round. In the southeast Atlantic adult albacore are caught down to 55°S by fisheries^141^. Moreover, several authors (e.g. ^110,124^), using catch rate statistics, have suggested that exchange of immature fish between the two oceans may occur during the austral summer. The Agulhas current might act as a facilitator of the transport of individuals from SWIO to SEAO. For the passage from SEAO to SWIO, the temperature should act as a barrier to young of the year albacore. On the other hand, adult individuals have larger temperature tolerances (between 14 and 21 °C, all oceans and information combined^142–149^), and be able to migrate from SEAO to SWIO. Yet, genetic analyses do not suggest a high level of migration, despite the apparent lack of physical barrier, such as in the case for bigeye tunas that are prevented from mutual penetration between Atlantic and Indo-Pacific^127^.

### Implications for stock assessments

One of the key elements required to improve the management of stocks is an enhanced understanding of the spatial delineation and dynamics of stocks, their connectivity, as well as the environmental drivers of those elements. Sustainable management of fisheries ideally requires the exploitation of one single population per stock^150^ to avoid the overexploitation and the risk of losing minority populations when various populations are managed as a single stock^31^. Furthermore there is an increasing requirement for traceability of fish, for consumer protection^151^ and regulatory enforcement^152^. Fisheries management is concerned about the demographically independent units’ identification^153,154^ and the stock boundaries representing restricted connectivity^150^. Units identified by fishery management, even if defined as biological entities^153^, might differ considerably from populations identified by genetic approaches^154^ that define the population structure according to the evolutionary history, the migrants, and the population size.

The present study suggests significant genetic differentiation between the southeast Atlantic and southwest Indian Oceans, supporting their demographic independence. The study of metal bioaccumulation and biogeochemical tracers in tuna muscle^155^ suggested movements of individuals between the two oceans. Distinct genetic clusters and morphometric differences, between and within Atlantic and Indian Oceans, are a result of a complex life cycle that includes migration and inter-ocean dispersal, yet with limited mating among groups. Juveniles and sub-adults observed in South Africa would mostly originate from the south Atlantic population as suggested by several authors^107,124,156^. The differentiation of southeast Atlantic and southwest Indian Oceans stocks has implications for the stock assessments of albacore tuna in the respective RFMOs, ICCAT and IOTC, as these need to reflect the most suitable geographical scale for stock management. Since the genetic differentiation reveals independence between the stocks, separate stock assessments are recommended, a strategy already adopted by the commissions’ working groups.

**Figure 5:**
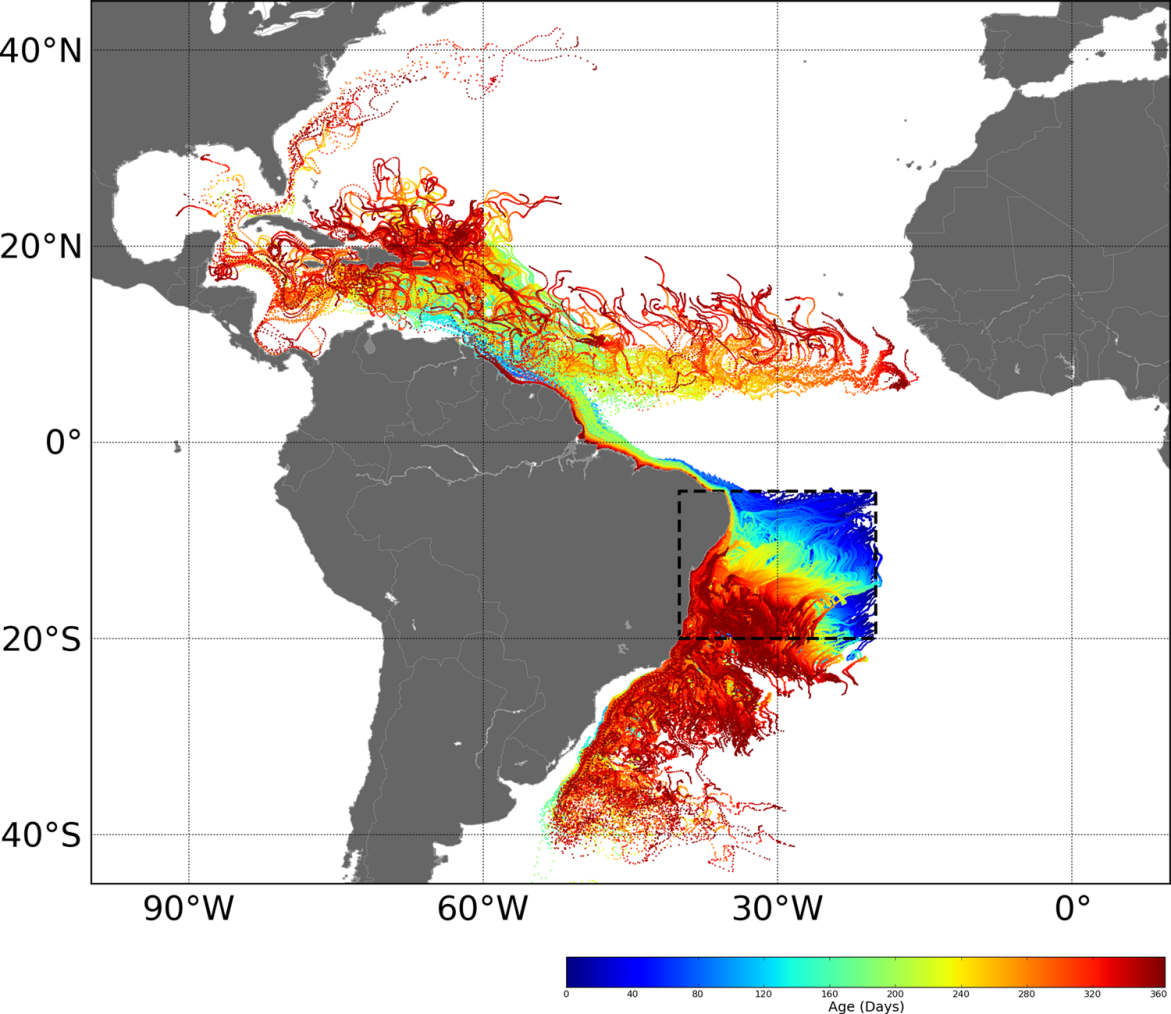
One-year long simulated passive drift trajectories for tuna larvae (and then small juveniles) released from a potential spawning area (delineated by dotted lines) situated offshore Brazil. Colors bar are in number of passive drift days.

According to the IOTC report^157^, the juvenile albacore caught off South Africa’s Atlantic coastline may originate in part from the northeast of Brazil. However, the simulation of passive drift does not support this assumption (Fig. 5), but revealed a potential pathway to an important fishing area in the North Atlantic. An increase of sampling localities with a clear strategy targeting spawning versus feeding grounds in both oceans would certainly help to improve the understanding of the grain size at which sub-structure develops among the albacore populations in the South Atlantic and Indian Oceans. For example, additional sampling in the eastern Indian Ocean should clarify the existence of two albacore populations in this ocean delimited by the 90°E meridian as suggested in different studies (e.g.^107,124,158^). Moreover, the present study sampled a small area of the southeast Atlantic Ocean that may be potentially separated from the rest of the south Atlantic. Sampling the northeast and northwest of the South Atlantic may help to further investigate the Atlantic origin of albacore caught off South Africa.

All of these results encourage further studies on the stock delineation of albacore in both oceans and both hemispheres, using different methods that are informative across nested time scales (direct tagging, biochemical markers, population modelling, multi-generation dispersal prediction, population assignment genetic analysis, Bayesian reconstruction of demography through genome scan data).

Our results provide a considerable degree of resolution concerning differentiation and levels of migration between both oceans, but there are still areas of uncertainty. Because genetic differentiation is the result of both historical processes and present-day gene flow, further investigation will be necessary to disentangle processes operating at different spatial and time scales. Collaborations between researchers and RFMOs provide promising avenues for enhancing our understanding of ecological and evolutionary mechanisms underlying population structure and connectivity of marine species, and information essential for their conservation and management. Inferring genetic connectivity and spatial genetic structure is difficult in large populations of tuna species that exhibit high fecundity and dispersal capabilities, resulting in low levels of genetic differentiation that prevent the reliable use of Bayesian reconstruction to disentangle long-term from contemporary connectivity patterns. Application of high-power genomic techniques (e.g. RAD or DART sequencing with mixed-stock analysis) could resolve the subtle boundaries and the sub-population structure.

Neutral SNPs and SNPs under selection can be used to identify discrete albacore populations under fine-grained resolution. Such approaches are likely to be routinely implemented in the future to discriminate tuna stocks, check their demographic status, diagnose illegal trade, and develop more sustainable management measures (as recommended for yellowfin tuna by^159^).

## Supplementary information

Supplementary Information 1.

Supplementary Information 2.

## Data Availability

All relevant data are within the published paper and its Supporting Information files. The dataset can be download at https://wwz.ifremer.fr/lareunion/Production-scientifique/Jeux-de-donnees or https://doi.org/10.17882/61552.
