# Supplementary material for: Connectivity and population structure of albacore tuna across southeast Atlantic and southwest Indian Oceans inferred from multidisciplinary methodology
Source: Sci Rep. 2020 Sep 24;10:15657. doi: 10.1038/s41598-020-72369-w (PMC7519111; doi:10.1038/s41598-020-72369-w)
Supplement: Supplementary file 1 — Supplementary Information 1. [file 41598_2020_72369_MOESM1_ESM.pdf]

Connectivity and population structure of albacore tuna across southeast Atlantic and southwest Indian Oceans inferred from multidisciplinary methods

Natacha Nikolic, Iratxe Montes, Maxime Lalire, Alexis Puech, Nathalie Bodin, Sophie Arnaud-Haond, Sven Kerwath, Emmanuel Corse, Philippe Gaspar, Stéphanie Hollanda, Jérôme Bourjea, Wendy West, Sylvain Bonhommeau

SUPPLEMENTARY MATERIAL

Appendix 1. Sample size per study group – genetic samples, fork length (FL), and round weight measurements. Four different geographic regions grouped by sampling periods (A, B, C, D) and sampling periods separate (A1, A2, B1, B2, C1, C2, D1, D2) (see Figure 1). Potential strategy (spawning and/or feeding) determined from scientific literature (e.g. Nikolic e al. 2016) and our study on reproduction stages and stomach contents in the GERMON project (e.g. Nikolic et al. IFREMER report 2015: https://archimer.ifremer.fr/doc/00293/40461/). (-) No information available.

Regions	Periods	Months	Potential strategy	Years	Genetic samples	Length samples	Weight samples
A	1	November	Spawning	2013	236	233	20
	2	April-August	Feeding	2014	230	228	216
	Other	All month		2001-2013	0	232	35
	sub-total				466	693	271
B	2	June-July	Feeding	2013	233	240	240
	2 (called 1 in database)	April-May	Feeding (also spawning in macroscopy stage)	2014	233	245	245
	sub-total				466	485	485
C	1	November-January	Spawning and feeding (literature)	2013-2014	323	327	131
	2	April-May	Feeding	2014	276	276	172
	sub-total				599	603	303
D	1	January	-	2014	157	157	0
	2	June	-	2014	191	191	0
	sub-total				348	348	0
	Total				1874	2129	1059

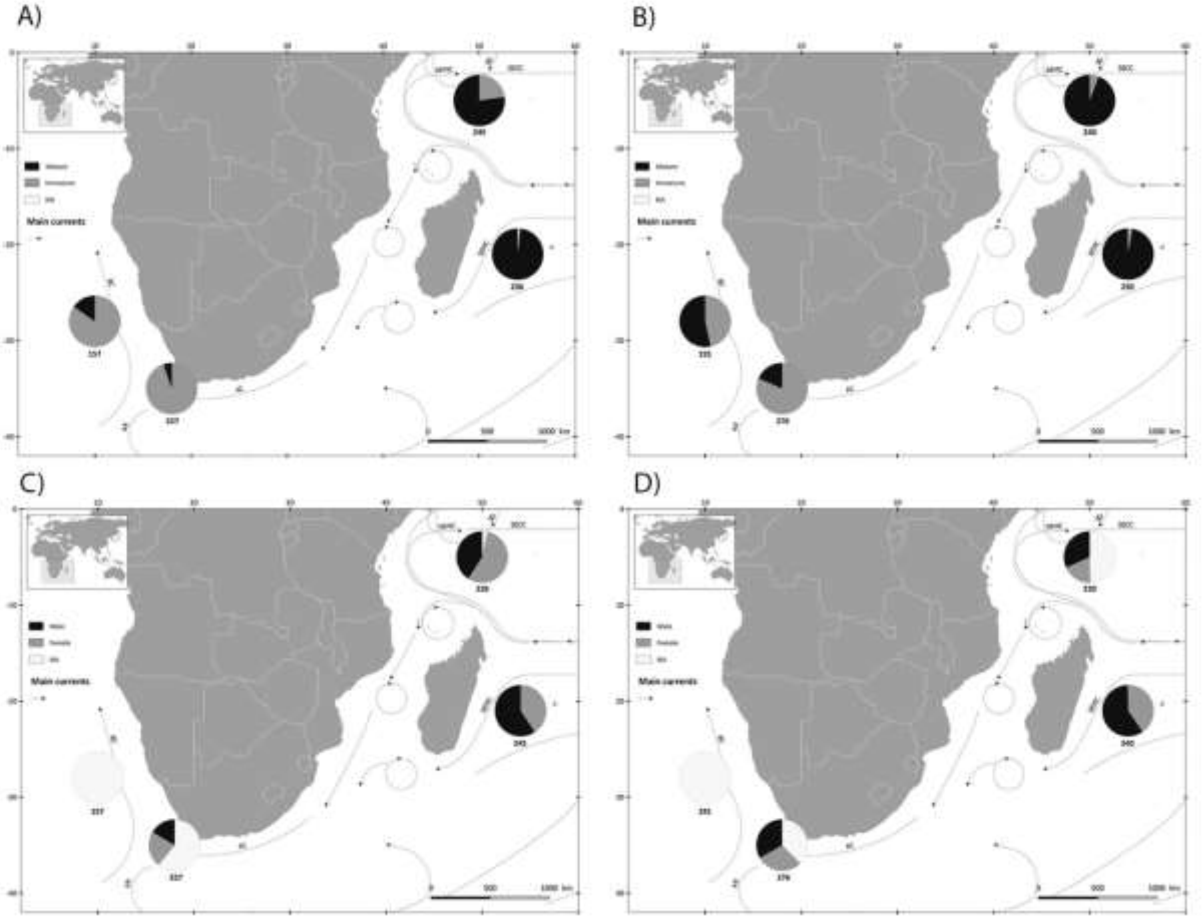

Appendix 2. Individual number and proportion of assumed mature and immature (from body length) albacore in austral summer (A) and winter (B) per sampling region (black – mature; grey – immature; white – unknown). Number and proportion of adult female and male albacore in austral summer (C) and winter (D) per sampling region (black – male; grey – female; white – unknown).

Appendix 3. Characteristics of 52 microsatellite markers for albacore (*Thunnus alalunga*). Number of individuals (Nind). Number of alleles (Na). Expected (He), unbiased Nei's (1978) expected (Hnb) and observed (Ho) heterozygosity. Significant homozygote and heterozygote excess (P<0.05) in bold. Polymorphism information content (PIC). *P* value for deviations from Hardy-Weinberg equilibrium (HWE) with exact tests by permutations and (/) Bonferroni correction: ns=not significant, * P<0.05, ** P<0.01, *** P<0.001. Null allele frequency (Fnull). Inbreeding coefficient (F_{IS}). Significant values are highlighted in bold (P<0.05) for heterozygote excess, Fnull, and Fis. Probability of identity (PI). Probability of exclusion (PE1, single parent; PE2, a second parent given a first parent assigned; PE3, a pair of parents). Number of repeated genotypes (Nrep and percentage (%) of the repeat number of individuals genotyped for each loci). Significant natural selection (alpha value). Genotyping error rate per allele, E1 referring to allelic dropout rate and E2 to the false allele rate, and the 95% confidence interval (CI). The last column corresponds to the markers selected.

																Genotyping error rate		Reference	Panel select
Locus	Nind	Na	He	Hnb	Ho	PIC	HWE	Fnull	*F*IS	PI	PE1	PE2	PE3	Nrep (%)	Significant alpha value	E1 (CI 95%)	E2 (CI 95%)		
ThuAla-mt-01	1481	27	**0.92**	**0.92**	**0.56**	0.92	***/***	**0.19**	**0.40**	0.01	0.72	0.84	0.96	10(73)		0.00 (-0.00-0.22)	0.00 (0.00-0.09)	Nikolic et al. 2015	
ThuAla-mt-02	1635	38	**0.92**	**0.92**	**0.88**	0.91	**/ns	0.02	**0.04**	0.01	0.71	0.83	0.95	11(81)	**-1.1650**	0.00 (-0.00-0.09)	0.00 (-0.00-0.07)	Nikolic et al. 2015	
ThuAla-mt-03	1598	37	**0.92**	**0.92**	**0.46**	0.92	***/***	**0.23**	**0.50**	0.01	0.73	0.84	0.96	12(92)		0.33 (-0.02-0.68)	0.00 (0.00-0.08)	Nikolic et al. 2015	
ThuAla-mt-04	1719	25	**0.91**	**0.91**	**0.70**	0.91	***/***	**0.11**	**0.23**	0.01	0.69	0.82	0.95	13(100)		0.00 (-0.00-0.42)	0.00 (0.00-0.06)	Nikolic et al. 2015	
ThuAla-mt-05	1714	8	**0.58**	**0.58**	**0.55**	0.52	***/ns	0.02	**0.05**	0.23	0.18	0.33	0.49	13(100)		0.00 (0.00-0.21)	0.00 (0.00-0.06)	Nikolic et al. 2015	x
ThuAla-mt-06	1793	13	**0.69**	**0.69**	**0.65**	0.63	***/**	0.02	**0.06**	0.15	0.27	0.44	0.62	13(100)		0.00 (0.00-0.07)	0.00 (0.00-0.06)	Nikolic et al. 2015	x
ThuAla-mt-07	1803	20	**0.67**	**0.67**	**0.65**	0.62	***/ns	0.01	**0.03**	0.16	0.26	0.43	0.61	13(100)		0.00 (0.00-0.07)	0.00 (0.00-0.06)	Nikolic et al. 2015	x
ThuAla-mt-08	1782	17	**0.84**	**0.84**	**0.73**	0.82	***/***	0.05	**0.13**	0.04	0.52	0.69	0.86	13(100)		0.00 (-0.00-0.06)	0.00 (0.00-0.06)	Nikolic et al. 2015	x
ThuAla-mt-09	1793	16	**0.77**	**0.77**	**0.68**	0.73	***/***	0.05	**0.12**	0.09	0.37	0.55	0.73	13(100)		0.00 (-0.00-0.06)	0.00 (0.00-0.06)	Nikolic et al. 2015	x
ThuAla-mt-10	1725	53	**0.87**	**0.87**	**0.80**	0.86	**/***	0.02	**0.07**	0.03	0.59	0.74	0.91	13(100)		0.00 (-0.00-0.07)	0.00 (0.00-0.06)	Nikolic et al. 2015	x
ThuAla-mt-11	1334	3	**0.23**	**0.23**	**0.19**	0.21	***/***	0.05	**0.17**	0.61	0.03	0.11	0.19	3(19)	**2.3182**	0.00 (0.00-0.08)	0.00 (0.00-0.06)	Nikolic et al. 2015	
ThuAla-mt-12	1665	12	**0.57**	**0.57**	**0.54**	0.54	*/ns	0.02	**0.04**	0.21	0.19	0.37	0.57	12(88)		0.00 (-0.00-0.13)	0.00 (-0.00-0.07)	Nikolic et al. 2015	x
ThuAla-mt-13	1842	10	**0.51**	**0.51**	**0.46**	0.48	***/***	0.03	**0.10**	0.27	0.15	0.31	0.50	13(100)		0.00 (0.00-0.15)	0.00 (0.00-0.06)	Nikolic et al. 2015	x
ThuAla-mt-14	1783	20	**0.84**	**0.84**	**0.81**	0.83	**/ns	0.01	**0.03**	0.04	0.53	0.70	0.87	13(100)		0.00 (-0.00-0.07)	0.00 (-0.00-0.06)	Nikolic et al. 2015	x
ThuAla-mt-15	1810	13	**0.62**	**0.62**	**0.59**	0.57	***/ns	0.02	**0.05**	0.19	0.21	0.38	0.56	13(100)		0.00 (-0.00-0.11)	0.00 (0.00-0.06)	Nikolic et al. 2015	x
ThuAla-mt-16	1654	25	**0.88**	**0.88**	**0.80**	0.87	***/***	0.03	**0.09**	0.03	0.61	0.76	0.92	12(92)		0.00 (0.00-0.07)	0.00 (0.00-0.06)	Nikolic et al. 2015	x
ThuAla-mt-17	885	21	**0.72**	**0.72**	**0.19**	0.67	***/***	**0.30**	**0.74**	0.12	0.31	0.49	0.67	6(42)		0.70 (0.03-1.81)	0.00 (-0.00-0.22)	Nikolic et al. 2015	
ThuAla-mt-18	1769	26	**0.89**	**0.89**	**0.76**	0.89	***/***	**0.07**	**0.15**	0.02	0.66	0.79	0.93	13(96)		0.00 (-0.00-0.06)	0.00 (0.00-0.06)	Nikolic et al. 2015	
ThuAla-mt-19	1403	16	**0.69**	**0.69**	**0.28**	0.65	***/***	**0.25**	**0.60**	0.14	0.29	0.45	0.64	11(85)		0.00 (0.00-0.14)	0.00 (0.00-0.07)	Nikolic et al. 2015	
ThuAla-mt-20	1641	58	**0.94**	**0.94**	**0.66**	0.94	***/***	**0.14**	**0.30**	0.01	0.80	0.89	0.98	13(96)		0.00 (-0.00-0.12)	0.00 (0.00-0.06)	Nikolic et al. 2015	
ThuAla-mt-21	1613	25	**0.84**	**0.84**	**0.40**	0.83	***/***	**0.24**	**0.52**	0.04	0.54	0.71	0.88	12(92)	**1.1119**	0.42 (-0.02-0.78)	0.00 (0.00-0.09)	Nikolic et al. 2015	
ThuAla-mt-22	1600	23	**0.85**	**0.85**	**0.49**	0.84	***/***	**0.19**	**0.42**	0.04	0.56	0.72	0.89	11(85)		0.41 (-0.98-0.84)	0.00 (0.00-0.10)	Nikolic et al. 2015	
ThuAla-mt-23	1781	19	**0.60**	**0.60**	**0.59**	0.57	*/ns	0.00	0.01	0.19	0.21	0.39	0.59	13(96)		0.00 (0.00-0.22)	0.00 (0.00-0.06)	Nikolic et al. 2015	x
ThuAla-mt-24	1824	18	0.85	0.85	0.83	0.84	ns/ns	0.00	**0.02**	0.04	0.55	0.71	0.88	13(100)		0.02 (0.00-0.06)	0.00 (0.00-0.06)	Nikolic et al. 2015	x

ThuAla-mt-25	1776	16	**0.76**	**0.76**	**0.73**	0.74	***/*	0.01	**0.04**	0.08	0.39	0.58	0.78	13(100)	**1.6184**	0.00 (0.00-0.12)	0.00 (-0.00-0.07)	Nikolic et al. 2015	
ThuAla-mt-26	1825	25	**0.72**	**0.72**	**0.67**	0.68	***/ns	0.02	**0.06**	0.12	0.32	0.49	0.68	13(100)		0.00 (-0.00-0.08)	0.00 (-0.00-0.06)	Nikolic et al. 2015	x
ThuAla-mt-27	1471	27	**0.85**	**0.85**	**0.44**	0.84	***/***	**0.22**	**0.48**	0.03	0.56	0.72	0.90	9(65)		0.00 (-0.00-0.25)	0.00 (0.00-0.09)	Nikolic et al. 2015	
ThuAla-mt-28	1716	40	**0.92**	**0.92**	**0.88**	0.91	*/ns	0.01	**0.04**	0.01	0.72	0.83	0.96	13(100)	**-1.5052**	0.00 (0.00-0.06)	0.00 (-0.00-0.06)	Nikolic et al. 2015	
ThuAla-mt-29	1775	32	**0.83**	**0.83**	**0.80**	0.82	**/ns	0.01	**0.04**	0.04	0.52	0.69	0.87	13(100)	**-1.4409**	0.00 (-0.00-0.06)	0.00 (-0.00-0.06)	Nikolic et al. 2015	
ThuAla-mt-30	1790	24	**0.86**	**0.86**	**0.84**	0.85	*/ns	0.01	**0.03**	0.03	0.58	0.73	0.90	13(100)		0.00 (0.00-0.00)	0.00 (0.00-0.06)	Nikolic et al. 2015	x
ThuAla-mt-31	1794	30	**0.91**	**0.91**	**0.88**	0.91	*/n	0.01	**0.03**	0.01	0.71	0.83	0.95	13(100)		0.00 (-0.00-0.07)	0.00 (-0.00-0.06)	Nikolic et al. 2015	x
ThuAla-mt-32	1794	23	0.67	0.67	0.68	0.66	ns/ns	0.00	-0.01	0.13	0.30	0.49	0.71	13(100)		0.00 (-0.00-0.08)	0.00 (0.00-0.06)	Nikolic et al. 2015	x
ThuAla-mt-33	1634	43	**0.96**	**0.96**	**0.91**	0.95	***/*	0.02	**0.05**	0.00	0.84	0.91	0.99	10(77)	**1.1038**	0.00 (0.00-0.07)	0.00 (0.00-0.07)	Nikolic et al. 2015	
ThuAla-mt-34	1740	21	**0.84**	**0.84**	**0.81**	0.83	*/ns	0.01	**0.04**	0.04	0.53	0.70	0.87	13(100)		0.00 (-0.00-0.07)	0.00 (-0.00-0.06)	Nikolic et al. 2015	x
Tth12-29	1842	15	**0.62**	**0.62**	**0.62**	0.55	**/ns	0.00	0.01	0.21	0.21	0.36	0.52	13(100)		0.00 (-0.00-0.07)	0.70 (0.49-1.59)	Clark et al. 2004	x
tth1-31	1787	26	0.81	0.81	0.80	0.79	ns/ns	0.00	0.01	0.06	0.47	0.64	0.83	13(100)		0.19 (0.09-0.33)	0.70 (0.57-2.85)	Clark et al. 2004	x
tth14	1755	12	**0.61**	**0.61**	**0.58**	0.54	***/ns	0.02	**0.06**	0.22	0.20	0.34	0.50	13(100)		0.34 (0.17-0.56)	0.70 (0.47-1.08)	Clark et al. 2004	x
tth157	1794	11	**0.57**	**0.57**	**0.58**	0.54	*/ns	0.00	-0.01	0.22	0.18	0.35	0.54	13(100)		0.05 (0.00-0.14)	0.70 (0.60-1.26)	Clark et al. 2004	x
Tth16-2	1561	9	**0.74**	**0.74**	**0.61**	0.69	***/***	0.05	**0.18**	0.11	0.33	0.50	0.68	13(100)		0.19 (0.09-0.32)	0.70 (0.59-2.79)	Clark et al. 2004	x
Tth17	1068	38	**0.88**	**0.88**	**0.36**	0.87	***/***	**0.27**	**0.59**	0.02	0.63	0.77	0.93	13(69)	**1.2765**	0.04 (0.00-0.12)	0.70 (0.61-1.19)	Clark et al. 2004	
tth178	1700	15	0.84	0.84	0.83	0.82	ns/ns	0.00	0.01	0.05	0.51	0.68	0.85	13(100)		0.00 (-0.00-0.05)	0.70 (0.62-2.80)	Clark et al. 2004	x
Tth185	1820	37	**0.93**	**0.93**	**0.92**	0.93	*/ns	0.00	**0.02**	0.01	0.76	0.87	0.97	13(100)	**-1.2346**	0.16 (0.07-0.29)	0.70 (0.58-2.51)	Clark et al. 2004	
Tth-21	1850	5	**0.53**	**0.53**	**0.52**	0.44	***/ns	0.02	0.02	0.32	0.14	0.24	0.37	13(100)		0.07 (0.00-0.18)	0.70 (0.61-1.46)	McDowell et al. 2002	x
tth226	1791	37	**0.93**	**0.93**	**0.88**	0.92	**/***	0.02	**0.05**	0.01	0.74	0.85	0.96	13(100)		0.00 (0.00-0.64)	0.70 (0.09-1.81)	Clark et al. 2004	x
Tth254	1786	43	**0.94**	**0.94**	**0.88**	0.94	***/***	0.03	**0.06**	0.01	0.78	0.88	0.97	13(100)		0.13 (0.00-0.50)	0.70 (0.30-2.18)	Clark et al. 2004	x
tth4	1714	60	0.95	0.95	0.94	0.95	ns/ns	0.00	**0.02**	0.00	0.83	0.90	0.98	13(100)	**-1.327**	0.05 (0.00-0.22)	0.70 (0.42-1.26)	Clark et al. 2004	
Tth-5	1760	34	**0.90**	**0.90**	**0.78**	0.89	***/***	0.05	**0.13**	0.02	0.67	0.80	0.94	13(100)		0.08 (0.022-0.17)	0.70 (0.60-1.53)	McDowell et al. 2002	x
tth62	1797	21	**0.78**	**0.78**	**0.74**	0.76	*/ns	0.01	**0.05**	0.07	0.42	0.60	0.79	13(100)		0.03 (0.00-0.13)	0.70 (0.52-3.50)	Clark et al. 2004	x
Ttho-1	1808	12	**0.62**	**0.62**	**0.60**	0.58	*/ns	0.01	**0.03**	0.18	0.22	0.40	0.58	13(100)		0.21 (0.12-0.40)	0.70 (0.57-3.30)	Takagi et al. 1999	x
Ttho-4	1681	35	0.88	0.88	0.84	0.87	ns/ns	0.01	**0.04**	0.02	0.63	0.77	0.93	13(92)		0.09 (-1.38-2.12)	0.17 (-0.02-1.90)	Takagi et al. 1999	x
Ttho-6	1614	19	**0.72**	**0.72**	**0.64**	0.70	***/***	0.04	**0.10**	0.10	0.34	0.53	0.74	13(100)		0.01 (-0.02-2.25)	0.55 (-1.51-2.62)	Takagi et al. 1999	x
Ttho-7	1755	23	**0.88**	**0.88**	**0.81**	0.87	***/***	0.03	**0.08**	0.03	0.61	0.76	0.91	13(100)	**1.6474**	0.05 (-0.84-1.90)	0.67 (-0.01-1.39)	Takagi et al. 1999	
Average	1689	24.54	0.78	0.78	0.67	0.76			0.14	0.09	0.48	0.63	0.78						**Total :** 32

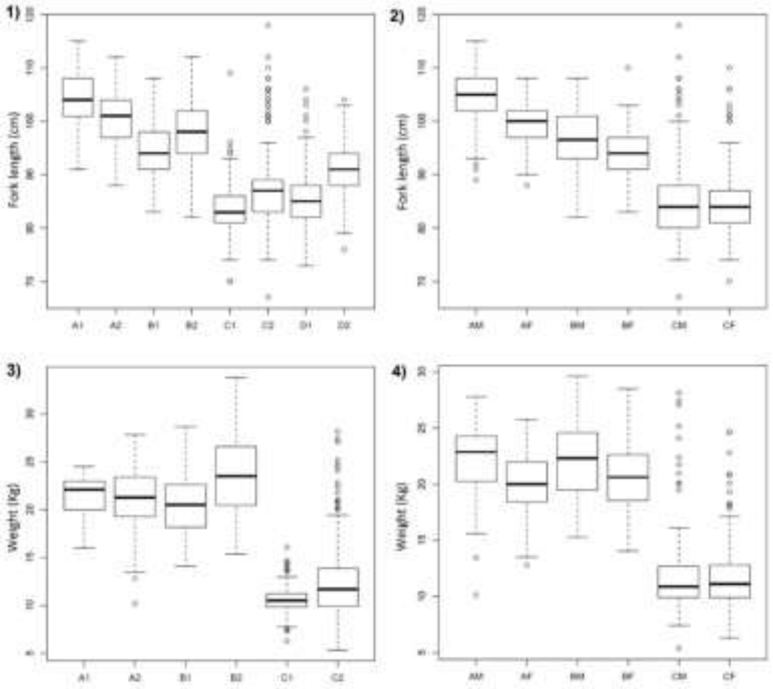

Appendix 4. Boxplot of albacore fork length (cm) (1 and 2) and whole weight (kg) (3 and 4) according to the regions (A, B, C, and D), periods (1 and 2) and sex (M = male, F = female). Four geographic regions, sampling periods grouped (A, B, C, D) and the sampling periods separate (A1, A2, B1, B2, C1, C2, D1, D2) (see Figure 1).

Appendix 5. Length-weight relationships of albacore tuna according the equation Weight (Kg) = a*FL^b from non-linear least squares (NLS) per geographic regions and sexes. n is the number of individuals, a a constant, b the allometric coefficient, R^2 the coefficient of determination, and Std. Error the standard error. The last column summarizes the Analysis of Covariance (ANCOVA) with the linear model (General Linear Model, GLM) between geographic regions (A, B, C, D) (see Figure 1).

Region	Sex	n	a	Std. Error(a)	b	Std. Error(b)	R^2	Analysis of covariance
**A**	F	107	$1.7415.10^{-5}$	$1.1013.10^{-5}$	3.0437	$1.3774.10^{-1}$	0.8304	Intercepts significant
	M	127	$3.2139.10^{-5}$	$1.8814.10^{-5}$	2.9044	$1.2620.10^{-1}$	0.8221	
**B**	F	182	$1.1214.10^{-4}$	$4.5330.10^{-5}$	2.6681	$8.8788.10^{-2}$	0.8364	Slopes and intercepts no significant
	M	176	$6.5699.10^{-5}$	$2.2731.10^{-5}$	2.7835	$7.5497.10^{-2}$	0.8893	
**C**	F	154	$2.1916.10^{-5}$	$6.4420.10^{-6}$	2.9656	$6.5569.10^{-2}$	0.9157	Slopes and intercepts no significant
	M	145	$1.9431.10^{-5}$	$5.8714.10^{-6}$	2.9921	$6.7080.10^{-2}$	0.9100	
**A-B**	F	289	$8.6863.10^{-4}$	$4.1654. 10^{-4}$	2.2077	$1.0501.10^{-1}$	0.6153	Slopes and intercepts no significant
	M	303	$1.6898.10^{-3}$	$7.0701.10^{-4}$	2.0629	$9.0831.10^{-2}$	0.6448	
**A-B-C**	F	443	$5.9820.10^{-6}$	$2.2672e.10^{-6}$	3.2888	$8.3227.10^{-2}$	0.8139	Slopes and intercepts significant
	M	448	$1.6173.10^{-5}$	$5.2244.10^{-6}$	3.0651	$7.0318.10^{-2}$	0.8463	

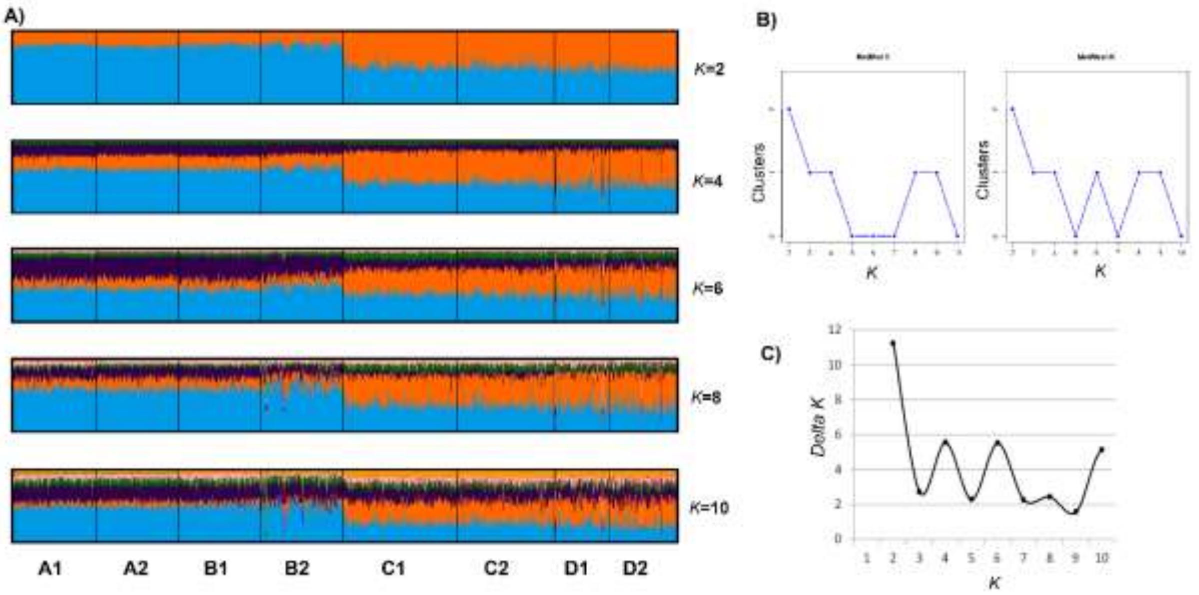

Appendix 6. Analysis of genetic population structure of albacore using individual Bayesian clustering in STRUCTURE for all populations and for the clusters *k* = 2; *k* = 4; *k* = 6; *k* = 8; *k* = 10; and *k* = 13 (A). In the right corner, (B) the Puechmaille (2016) and (C) the Evanno *et al.* (2005) methods – mean ln(k) and mean Δk (Delta K) over 5 runs estimated for the number of clusters (*k*) ranging from 1 to 14. Considering the denomination sampling case T2 (regions A1, A2, B1, B2, C1, C2, D1, and D2). Best *k* = 2.

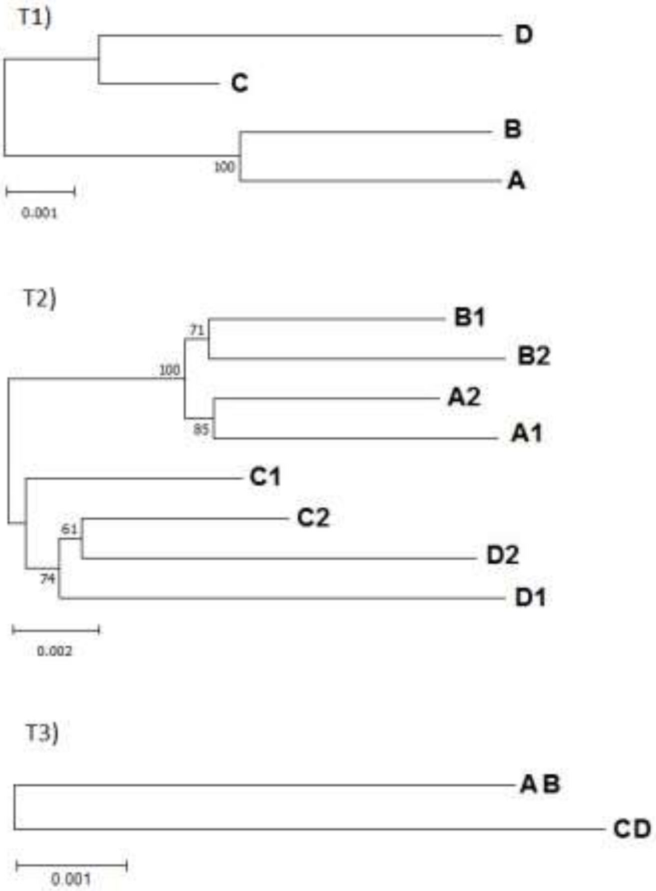

Appendix 7. Differentiation of albacore samples over 32 microsatellite loci considering the three scenario (T1, T2, and T3) with NJ tree based on *D*SW genetic distance. The percentage of replicate trees in which the associated taxa clustered together in the bootstrap test (100 000 replicates) are shown next to the branches.

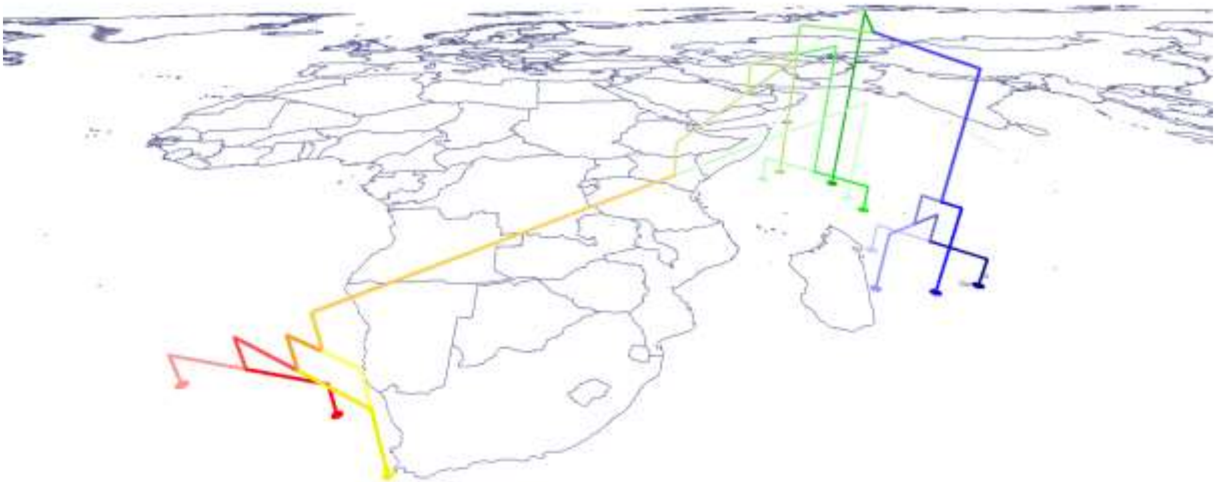

Appendix 8. 3D geophylogeny tree using NJ tree from Dsw genetic distances of albacore tuna samples over 32 microsatellite loci. Region A (blue colours), region B (green colours), region C (yellow colours), and D (red colours). Intensity of colours do not represent the link between the sites, but distinguish among geographical sites in the same region.

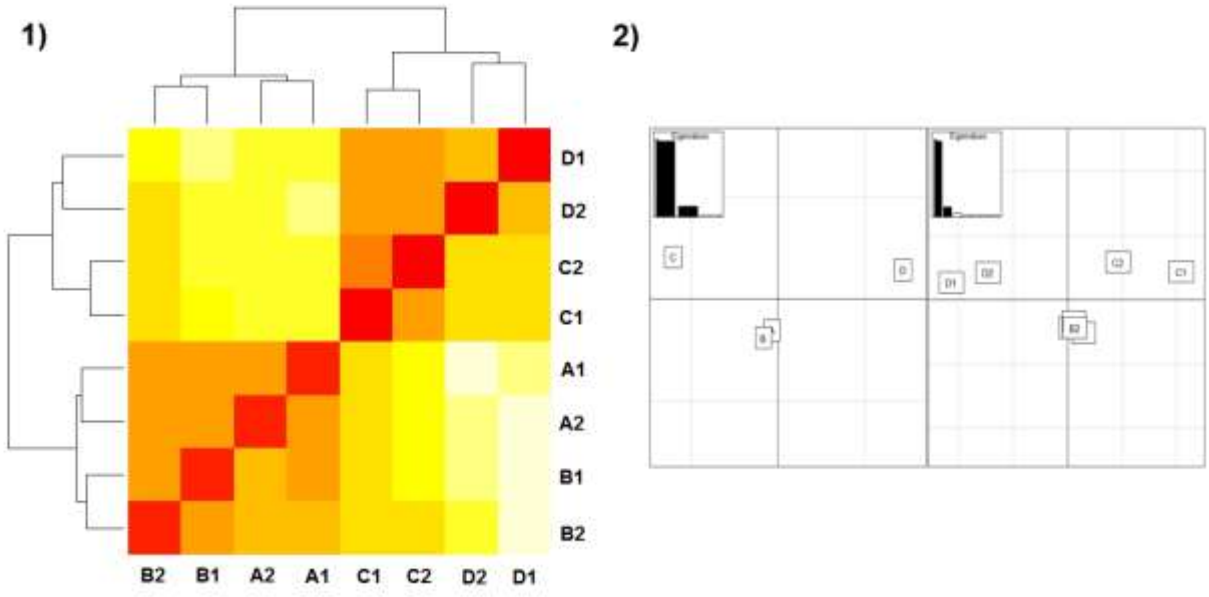

Appendix 9. Differentiation of albacore samples over 32 microsatellite loci considering cases, T1 and/or T2.
(1) Heatmap of pairwise F_{ST} values with a dendrogram.
(2) Principal Component Analysis on allelic frequencies.

A)

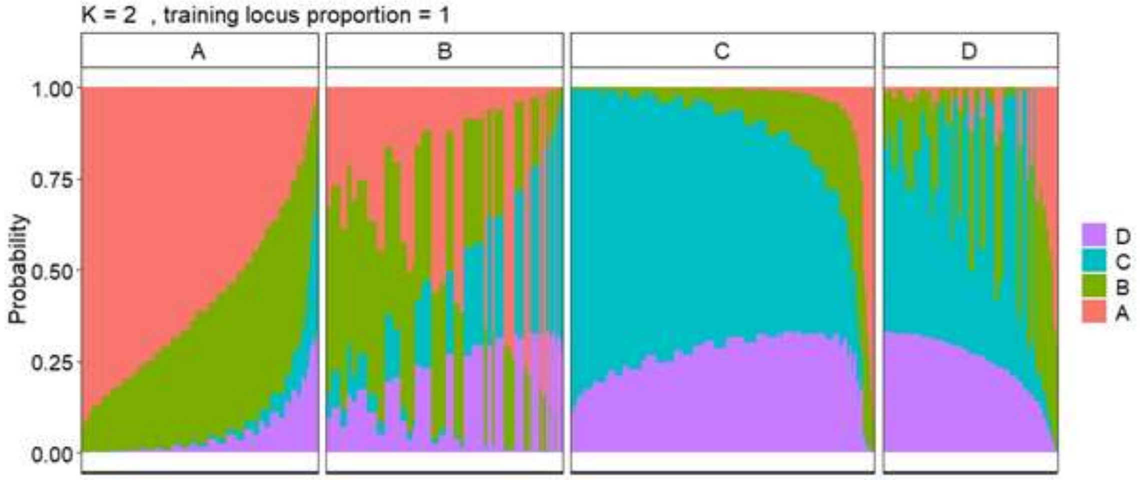

B)

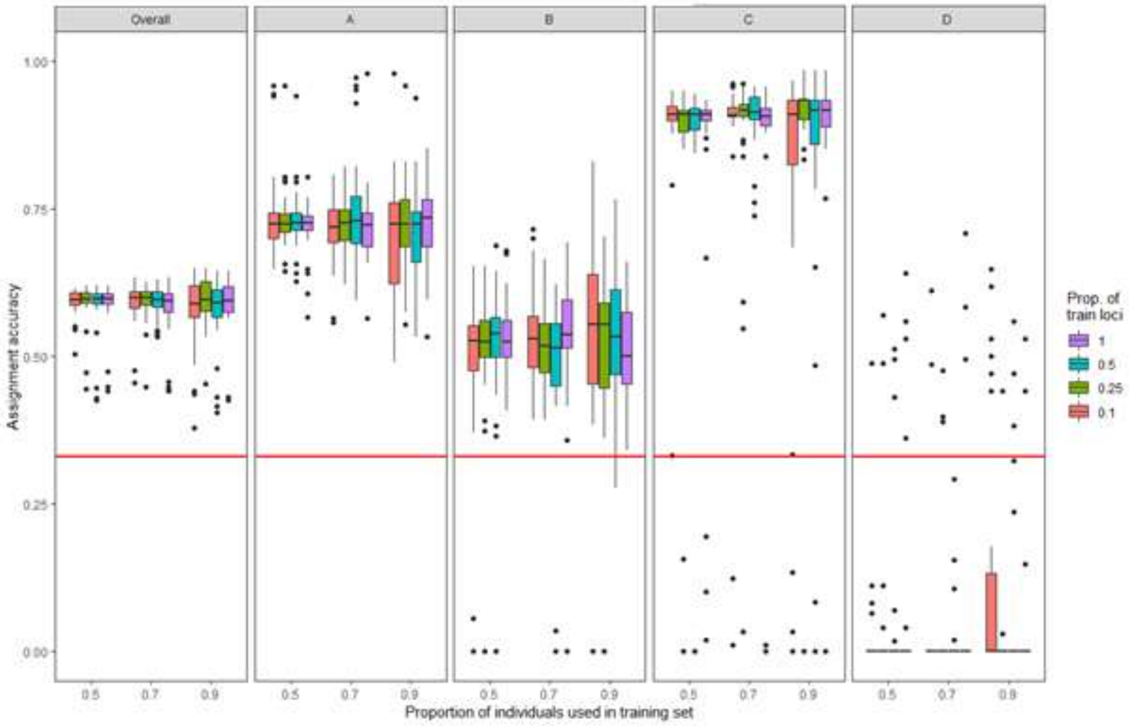

Appendix 10. A) Membership probability with results estimated via 2-fold cross-validation using overall loci and morphometric data. Individuals are sorted based on the probability of assignment to their original populations. **B)** Assignment accuracies estimated via Monte-Carlo cross-validation with overall loci and morphometric data for four hypothetical populations (A, B, C, and D). The left figure is the assignment on overall individuals and without hypothetical population. Red horizontal lines indicate null assignment rate.

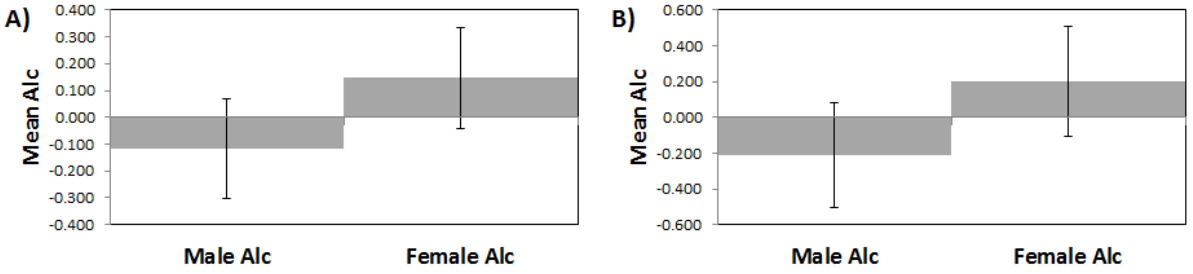

Appendix 11. Assignment Index correction (AIc) values for each sex (Male and Female) with the individuals of the genetic cluster from southwest Indian Ocean (A) and genetic cluster from southeast Atlantic Ocean (B). Mean negative AIc values characterize individuals with a higher probability of being immigrants, whereas positive values characterize individuals with a lower probability of being migrants.

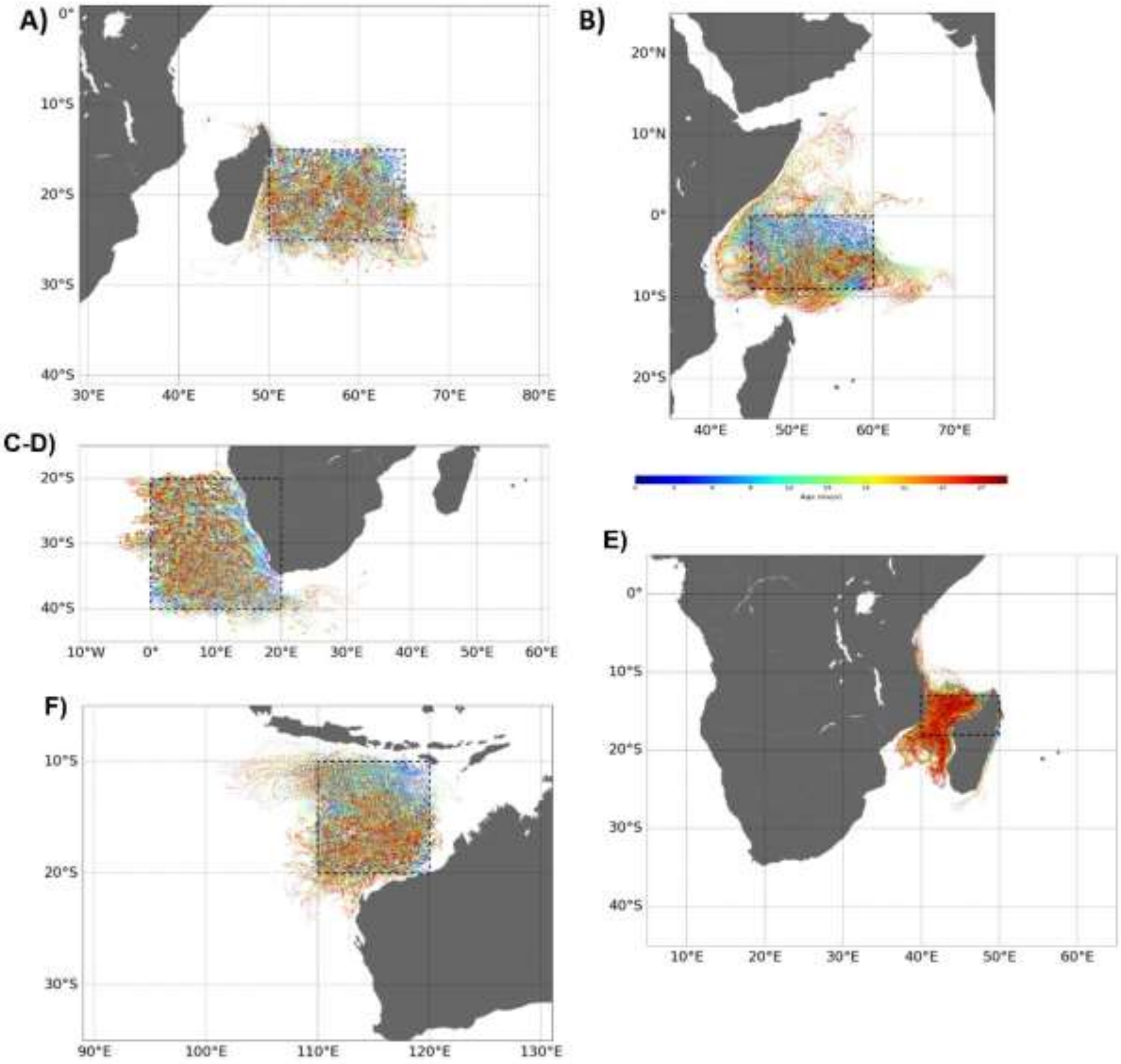

Appendix 12. One-month long simulated passive drift trajectories for tuna larvae (and then small juveniles) released from different potential spawning areas (delineated by dotted lines):

West Madagascar (A), North Madagascar (B), Mozambique Channel (E) and North Australia (F) Three-month long simulated passive drift trajectories are also shown for particles released in the southeast Atlantic (C-D).

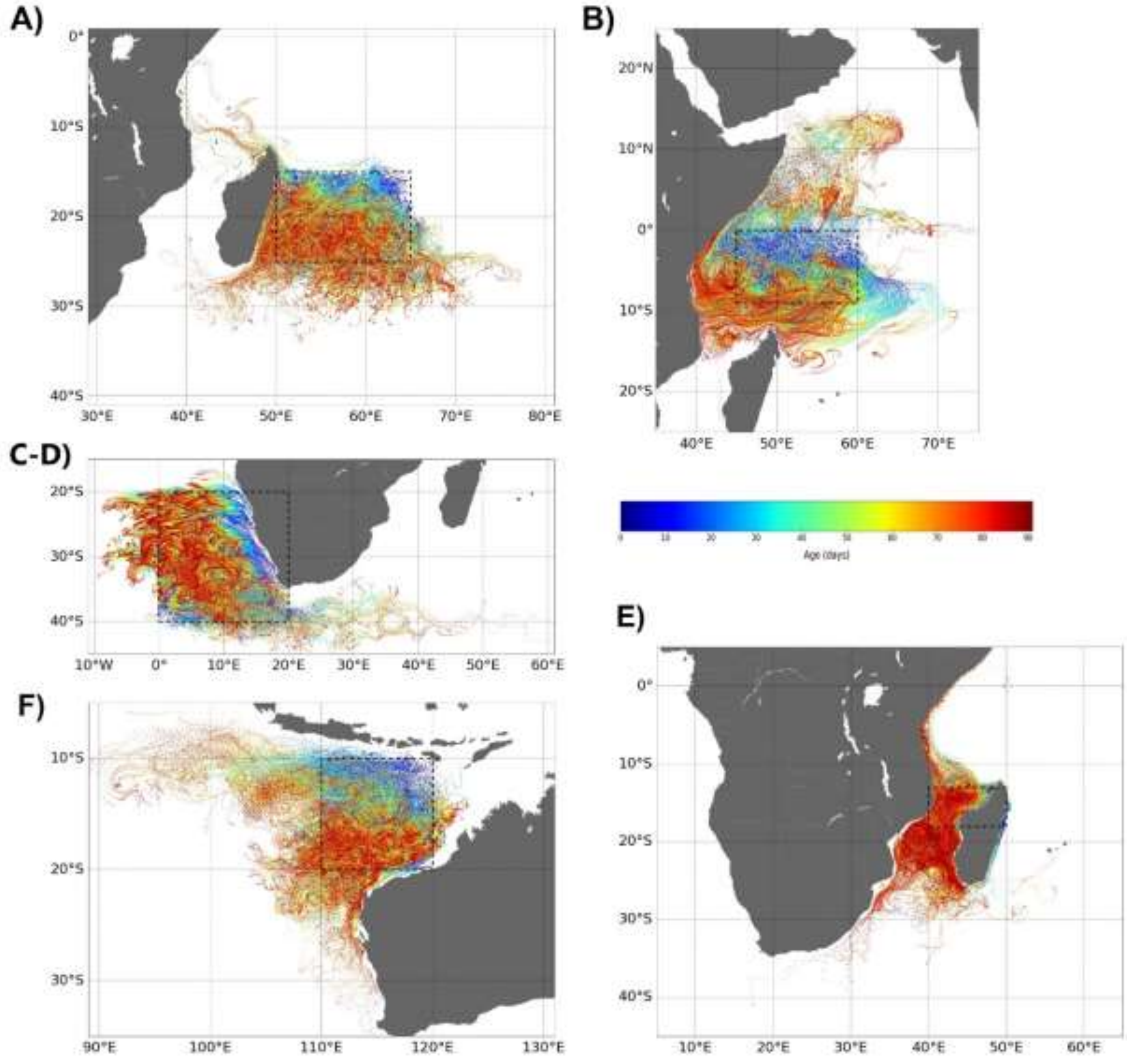

Appendix 13. Three-month long simulated passive drift trajectories for tuna larvae (and then small juveniles) released from different potential spawning areas (delineated by dotted lines): West Madagascar (A), North Madagascar (B), Mozambique Channel (E) and North Australia (F) Three-month long simulated passive drift trajectories are also shown for particles released in the southeast Atlantic (C-D).

Appendix 14. R square analysis at NJ and UPGMA tree with the selected panel of markers (32 microsatellites) for the three sampling cases. Here, the degree of fit of a tree to a matrix of genetic distances quantified with R^2 is higher with NJ than UPGMA for all genetic distances with the selected panel.

**Sampling case**	**Tree**	**Nei 1978**	**Nei 1987**	**Cavalli-Sforza 1967**	**Weir & Cockerham 1984**
Scenario T1	NJ	1	1	1	1
(A, B, C, D)	UPGMA	0.78	0.65	0.72	0.78
Scenario T2	NJ	0.96	0.58	0.65	0.96
(A1, A2, B1, B2, C1, C2, D1, D2)	UPGMA	0.78	0.97	0.98	0.78
Scenario T3	NJ	1	1	1	1
(AB, CD)	UPGMA	1	1	1	1

Appendix 15. Migration rates (*Nm*) among regions with scenario T1 and T3 according to Wright (1969) above the diagonal and private allele frequency after correction for size below the diagonal.

Scenario T1

Regions	A	B	C	D
A		8256.06	163.71	93.09
B	32.02		131.88	78.29
C	35.92	34.29		568.48
D	22.69	24.39	38.20	

Scenario T3

Regions	**AB**	**CD**
**AB**		121.37
**CD**	44.94	
