# Supplementary material for: Connectivity and population structure of albacore tuna across southeast Atlantic and southwest Indian Oceans inferred from multidisciplinary methodology
Source: Sci Rep. 2020 Sep 24;10:15657. doi: 10.1038/s41598-020-72369-w (PMC7519111; doi:10.1038/s41598-020-72369-w)
Supplement: Supplementary file 2 — Supplementary Information 2. [file 41598_2020_72369_MOESM2_ESM.pdf]

Connectivity and population structure of albacore tuna across southeast Atlantic and southwest Indian Oceans inferred from multidisciplinary methods

Natacha Nikolic, Iratxe Montes, Maxime Lalire, Alexis Puech, Nathalie Bodin, Sophie Arnaud-Haond, Sven Kerwath, Emmanuel Corse, Philippe Gaspar, Stéphanie Hollanda, Jérôme Bourjea, Wendy West, Sylvain Bonhommeau

SUPPLEMENTARY TEXT

(S1) Material and Methods:

(S1-a) Selection of microsatellite loci

A total of fifty-two microsatellite *loci* were analyzed in this study (Appendix 3). Thirty-four were directly developed in the target species *Thunnus alalunga* by Nikolic *et al.* (2015), whereas 18 markers were cross-amplified from other species from genus *Thunnus*. These comprised four microsatellite *loci* originally described in Pacific bluefin tuna, *T. orientalis* (Takagi *et al.* 1999), and 14 microsatellite markers originally characterized in Atlantic bluefin tuna, *T. thynnus* (McDowell *et al.* 2002; Clark *et al.* 2004).

Genetic variation levels from each of the 52 microsatellite markers were analyzed using GENETIX 4.05 (Belkhir *et al.* 1996) to determine levels of diversity (allelic richness, Na), expected heterozygosity (He), expected unbiased heterozygosity from Nei (Hnb), and observed heterozygosity (Ho). Estimates of homozygote and heterozygote excess zeros were calculated from the standard error in Pedant ($P < 0.05$) (Johnson and Haydon 2007). Polymorphism information content (PIC) was generated in Cervus (Kalinowski *et al.* 2007).

Departures from the expectations of Hardy-Weinberg equilibrium (HWE) were detected by exact tests and permutations (1000 000 chains and 100 000 steps) with ARLEQUIN v3.1 (Excoffier *et al.* 2005) and with Bonferroni correction using Cervus. Null allele frequency (Fnull) was estimated with INEst (Chybicki and Burczyk 2009) using the individual inbreeding model (i.e. Gibbs sampler method) ($P < 0.05$) (frequency was also controlled with GENEPOP version 4.7.2 (Rousset 2008; Rousset 2019) and was concordant), followed by MICRO-CHEKER (Van Oosterhout *et al.* 2004) in order to identify microsatellite markers with a significant amount of null alleles and remove them for posterior analyses. Linkage disequilibrium between all pairs of loci was performed using ARLEQUIN per permutation (10 000). F_{IS} (inbreeding coefficient) and its significance was estimated by permutation (10 000) using GENETIX, then also verified by INEst.

Probability of identity (PI) by *locus* was estimated using GenAlEx v6 (Peakall and Smouse 2006). PI is an advanced frequency-based analysis, also referred to as population match probability that provides an estimate of the average probability that two unrelated individuals will have the same multi-*locus* genotype. It indicates the statistical power of marker *loci*. Probability of exclusion (PE1, single parent; PE2, a second parent given a first parent assigned; PE3, a pair of parents) was estimated per *locus* using INest. Genotyping error rate per allele, E1 referring to allelic dropout rate and E2 to the false allele rate, and the 95% confidence interval (CI), was evaluated using Pedant with 10 000 permutations based on He with 13 individuals repeated genotypes. The number of these repeated genotypes (Nrep) and the percentages (%) of the total number of individuals repeated genotypes (13) for each *loci* was also estimated. All microsatellite *loci* were tested for natural selection using BAYESCAN 2.1 software (Foll and Gaggiotti 2008) with 100 000 iterations. The average mutation rate over *loci*

(u) were estimated using MSVAR (Beaumont 1999), running 16 x 10^8 (80 000 * 20 000) steps per Monte-Carlo Markov chain (MCMC).

A set of microsatellite markers retaining only the ones not showing evidence for null alleles or selection was used for population structure analyses (see selected panel, Appendix 3).

POWSIM v. 4.1 (Ryman and Palm 2006) was used to estimate whether the number of selected *loci* from classical analysis and their allele frequencies provided sufficient statistical power to detect significant genetic differentiation. Simulations were run using default parameter values for dememorizations (1000), batches (100) and iterations per batch (1000), and a range of different values of F_{ST} (0.0001–0.05) were tested by varying the number of generations of drift (t) while keeping the effective population size (Ne) constant at 4000 (range of value estimated from VarEff software, Nikolic and Chevalet 2014). The statistical power was estimated after 1000 replicates as the proportion of statistically significant tests ($P < 0.05$). The probability of obtaining false positives when the true $F_{ST} = 0$ was also obtained at generation $t = 0$ as a measure of α error rate.

Microsatellite analyses were performed on the selected panel of markers (Appendix 3) in each sample (Figure 1), considering the following scenarios: (T1) the sampling periods together (regions A, B, C, and D), (T2) the sampling periods separate (regions A1, A2, B1, B2, C1, C2, D1, and D2), and (T3) same ocean samples together (regions A-B and C-D). Finally, previously analysed reproductive stage (Dhurmeea *et al.* 2016) was taken into account in order to group individuals in full or not reproductive stage to help in understanding the structure in spawning grounds.

(S1-b) Additional data analysis

To measure the degree to which tree UPGMA (Sneath and Sokal 1973) or neighbor-joining (NJ) (Saitou and Nei 1987) better explains genetic relationships among populations, the R squared (R^2) of Kalinowski (2009) with all genetic distances available (Nei 1978; Nei 1987, Weir and Cockerham 1984; Cavalli-Sforza and Edwards 1967) was used by the TreeFit software (Kalinowski 2008). D_{SW} (Shriver et al. 1995) genetic distances are highly effective for recognizing population associations (Destro-Bisol *et al.* 2000; Pérez-Lezaun *et al.* 1997; by Crawford 2007) particularly with microsatellite loci and stepwise mutation models (SMM) (Shriver *et al.* 1995).

Network analysis was implemented in EDENETWORKS v2.18 (Kivelä *et al.* 2014) to construct a minimum-spanning tree (MST). An MST is the minimal network necessary to connect all populations in the sample. For this purpose, the program plots all populations (nodes) in a network graph with connections (edges) between all nodes. Each edge is weighted according to Goldstein genetic distance (Goldstein *et al.* 1995) and 1000 bootstrapping samples. Goldstein genetic distance was developed specifically for microsatellite markers and is based on the SMM of evolution (Rozenfeld *et al.* 2007). Network construction was based on genetic distance between populations with no further priors, and the layout of the MST was recalculated 10 times to test for possible alternative network shapes.

ARLEQUIN was used for the Analysis of Molecular Variance (AMOVA) employing the geographic areas as populations and genetic clusters found from previous analysis as groups.

Number of migrants (*Nm*) were estimated through the private alleles frequency with GENEPOP online (http://genepop.curtin.edu.au) and also according to Wright (1969) using GENETIX.

(S2) Results:

(S2-a) Morphology analysis

Table 1 presents the characteristics of length-weight relationships per region. The t-test showed that albacore from region B exhibit allometric growth ($p < 0.00005$) with an exponent parameter (b) between 2.67 and 2.83, with 95% confidence. Whereas, albacore individuals from A and C regions exhibited isometric growth (no presumption against the null hypothesis) with an exponent parameter (b) between 2.74 -3.03 and 2.84-3.06, respectively. South African samples (region C) are thus composed mainly of immature individuals with an allometric factor close to 3 (Table 1), suggesting isometric growth with body shape invariant with age.

(S2-b) Selection of microsatellite loci

Most of the 52 markers had a large number of alleles per *locus* (Na), ranging from 3 to 60 alleles (Appendix 3). He and Hnb varied from 0.23 to 0.96, and Ho from 0.19 to 0.94. The polymorphic information content (PIC) value averaged 0.76 (SD ± 0.17). Two markers presented low number of alleles (ThuAla-mt-11 and Tth-21) and one presented low heterozygosity (>0.5), low PIC, high PI, and low PE (ThuAla-mt-11). Probability of identity (PI) ranged from 0 to 0.61, and the probability of exclusion (PE1, PE2, PE3) from 0.03 to 0.99. Only 1 marker (ThuAla-mt-11) showed high probability of identity (PI>0.5), and 49 markers exhibited high probability of exclusion (PE>0.5).

A total of 11 markers had null alleles, as suggested by overall homozygote excess for most allele size classes. Stuttering might have resulted in scoring errors except for 4 out of these microsatellites (ThuAla-mt-19, ThuAla-mt-03, ThuAla-mt-04, and ThuAla-mt-01). These 11 *loci* with null alleles were discarded from the dataset.
A total of 11 markers gave significant results when tested for selection, with 5 markers for divergent selection (negative alpha values), and 6 markers for balancing selection (positive alpha values). A BLAST search revealed similarity of *locus* Ttho-7 (under divergent selection) to cpu-UA/3 MHC class I antigen gene (Genbank accession number AY008848.2) from the teleost fish *Ictalurus punctatus*. From these 11 *loci* putatively under selection, 3 were already discarded during the previous step (null alleles) and the other 8 were discarded for further analysis that assess neutral variation. Thus, for the markers (46 without and 27 with Bonferroni correction) that departed significantly from Hardy–Weinberg equilibrium, but showed no linkage disequilibrium, no presence of significant results for null alleles and no under selection (33 markers) were maintained in the dataset, since departure from HWE at a locus can result from the population or sample features and not from the locus itself (Wittke-Thompson *et al.* 2005). The selected markers have all frequency of null all inferior or equal to 0.05 (so very low) and are not significant. Additionally, the marker ThuAla-mt-11 was discarded due to its low number of alleles (3), heterozygosity (excepted 0.23; observed 0.19), PIC (0.21) and PE (0.03-0.19). Therefore, the set of markers for further analyses consisted of 32 highly polymorphic microsatellites (Appendix 3, see the last column).

Simulations indicated that the 32 loci dataset designed in this study allowed detecting significant differences among two well differentiated groups on each side of southern Africa, each of them corresponding to one ocean. Fine scale population genetic structure often needs a large number of polymorphic microsatellite markers and the panel dataset developed in this study is of higher quality corresponding to the general recommended number (Barker *et al.* 1993; Luikard and Cornuet 1998; SanCristobal *et al.* 2006; Nikolic *et al.* 2009). The high polymorphism of these markers set proves its usability in the characterization and evaluation of genetic diversity within and between albacore populations, and these markers constitute a useful tool for obtaining detailed information on the genetic diversity and structure of this species and investigating its evolutionary history.

(S2-c) Genetic data analysis

R square analysis revealed that NJ tree was better than UPGMA to summarize a genetic distance (Appendix 14).

Gene flow (number of migrants per generation, *N*m) analyses (Appendix 15) showed high values particularly within the defined genetic clusters, which is consistent with all structural and differentiation results; and the magnitude values are linked to geographical distance.

(S3) References:

Albaina A, Iriondo M, Velado I, Laconcha U, Zarraonaindia I, Arrizabalaga H, Pardo MA, Lutcavage M, Grant WS, Estonba A. 2013. Single nucleotide polymorphism discovery in albacore and Atlantic bluefin tuna provides insights into worldwide population structure. Animal Genetics, 44:678-692.

Barker JSF, Bradley DG, Fries R, Hill WG, Nei M, Wayne RK. 1993. An integrated global program to establish the genetic relationships among the breeds of each domestic animal species. Rome: FAO Animal Production and Health Paper, Report of a working group.

Beaumont MA. 1999. Detecting population expansion and decline using microsatellites. Genetics, 153:2013–2029.

Belkhir K, Borsa P, Chikhi L, Raufaste N, Bonhomme F. 1996. GENETIX, logiciel sous WindowsTM pour la génétique des populations. Laboratoire Génome, Populations, Interactions CNRS UMR 5000. Montpellier: Université de Montpellier II.

Cavalli-Sforza LL, Edwards AWF. 1967. Phylogenetic analysis: models and estimation procedures. Evolution, 21:550-570.

Chybicki IJ, Burczyk J. 2009. Simultaneous estimation of null alleles and inbreeding coefficients. Journal of Heredity, 100:106–113.

Çiftci Y, Okumuş I. 2002. Fish Population Genetics and Application of Molecular Markers to Fisheries and Aquaculture – I: Basic Principles of Fish Population Genetics. Turkish Journal Fisheries and Aquatic Sciences, 2:145-155.

Crawford MH. 2007. Anthropological Genetics: Theory, Methods and Applications. Cambridge: Cambridge University Press. Crawford MH. Edited by Michael H. Crawford.

Davies CA, Gosling EM, Was A, Brophy D, Tysklind N. 2011. Microsatellite analysis of albacore tuna (*Thunnus alalunga*): population genetic structure in the North-East Atlantic Ocean and Mediterranean Sea. Marine Biology, 158:2727–2740.

Destro-Bisol G, Boschi I, Caglià A, Tofanelli S, Pascali V, Paoli G, Spedini G. 2000. Microsatellite variation in Central Africa: an analysis of intra-populational and interpopulational genetic diversity. American Journal of Physical Anthropology, 112:319-337.

Dhurmeea Z, Zudaire I, Chassot E, Cedras M, Nikolic N, Bourjea J, et al. 2016. Reproductive Biology of Albacore Tuna (*Thunnus alalunga*) in the Western Indian Ocean. PLoS ONE 11(12): e0168605.doi:10.1371/journal.pone.016860

Foll M, OE Gaggiotti. 2008. A genome scan method to identify selected *loci* appropriate for both dominant and codominant markers: A Bayesian perspective. Genetics, 180: 977-993

Goldstein DB, Linares AR, Cavalli-Sforza LL, Feldman MW (1995) (1995b) Genetic absolute dating based on microsatellites and the origin of modern humans. Proc Natl Acad Sci USA 92:6723–6727.

Johnson PCD, Haydon DT. 2007. Maximum-likelihood estimation of allelic dropout and false allele error rates from microsatellite genotypes in the absence of reference data. Genetics, 175:827–842.

Kalinowski ST. 2009. How well do evolutionary trees describe genetic relationships between populations? Heredity, 102:506-513.

Kalinowski ST. 2008. Treefit. A computer program for evaluating how well evolutionary trees fit genetic distance data. http://www.montana.edu/kalinowski/Software/TreeFit.htm.

Kalinowski ST, Taper ML, Marshall TC. 2007. Revising how the computer program CERVUS accommodates genotyping error increases success in paternity assignment. Molecular Ecology, 16:1099–1106.

Kivelä M, Arnaud-Haond S, Saramäki J. 2014. EDENetwork: Ecological and Evolutionary Networks. Molecular Ecology Resources: http://becs.aalto.fi/edenetworks/. doi: 10.1111/1755-0998.12290.

Kumar S, Stecher G, and Tamura K. 2016. MEGA7: Molecular Evolutionary Genetics Analysis version 7.0 for bigger datasets. Molecular Biology and Evolution, 33:1870-1874.

Luikart G, Cornuet JM. 1998. Empirical evaluation of a test for identifying recently bottlenecked populations from alleles frequencies data. Conserv Biol 12:228–237.

Montes I, Iriondo M, Manzano C, Arrizabalaga H, Jimánez E, Pardo MA, Goñi N, Davies CA, Estonba A. 2012. Worldwide genetic structure of albacore (*Thunnus alalunga*) revealed by microsatellite DNA markers. Marine Ecology Progress Series, 471:183–191.

Nakadate M, Viñas J, Corriero A, Clarke S, Suzuki N, Chow S. 2005. Genetic isolation between Atlantic and Mediterranean albacore populations inferred from mitochondrial and nuclear DNA markers. Journal of Fish Biology, 66:1545-1557.

Nikolic N, Duthoy S, Destombes A, Bodin N, West W, Puech A, Bourjea J. 2015. Discovery of genome-wide microsatellite markers in Scombridae: a pilot study on albacore tuna. Plos One, 10(11):e0141830.

Nikolic N, Chevalet C. 2014. Detecting past changes of effective population size. Evolutionary Applications, (7):663-681.

Nikolic N, Fève K, Chevalet C, Høyheim B, Riquet J. 2009. A set of 37 microsatellite DNA markers for genetic diversity and structure analysis of Atlantic salmon *Salmo salar* populations. Journal of Fish Biology, 74:458–466.

Nei M. 1987. Molecular Evolutionary Genetics. Columbia University Press, New York.

Nei M. 1978. Estimation of average heterozygosity and genetic distance from a small number of individuals. Genetics, 89:583–590.

Okumuş I, Çiftci Y. 2003. Fish Population Genetics and Molecular Markers: II- Molecular Markers and Their Applications in Fisheries and Aquaculture. Turkish Journal of Fisheries and Aquatic Sciences, 3:51-79.

Pérez-Lezaun A, Calafell F, Mateu E, Comas D, Ruiz Pacheco R, Bertranpetit J. 1997. Microsatellite variation and the differentiation of modern humans. Human Genetics, 99:1-7.

Rousset F. 2008. Genepop'007: a complete reimplementation of the Genepop software for Windows and Linux. Molecular Ecology Resources 8:103–106.

Rousset F. 2019. Genepop Version 4.7.2. Available at: https://kimura.univ-montp2.fr/~rousset/Genepop4.7.pdf

Rozenfeld AF, Arnaud-Haond S, Hernández-García E et al. 2007. Spectrum of genetic diversity and networks of clonal populations Journal of the Royal Society Interface.

Ryman N, Palm S. 2006. POWSIM: a computer program for assessing statistical power when testing for genetic differentiation. Mol. Ecol. Notes, 6:600–602.

Saitou N, Nei M. 1987. The neighbor-joining method: A new method for reconstructing phylogenetic trees. Molecular Biology and Evolution, 4:406-425.

SanCristobal M, Chevalet C, Haley CS, Joosten R, Rattink AP, Harlizius B, Groenen MAM, Amigues Y, Boscher M-Y, Russell G, Law A, Davoli R, Russo V, Désautés C, Alderson L, Fimland E, Bagga M, Delgado JV, Vegapla JL, Martinez AM, Ramos M, Glodek P, Meyer JN, Gandini GC, Matassino D, Plastow GS, Siggens K, Laval G, Archibald AL, Milan D, Hammond K, Cardellino R. 2006. Genetic diversity within and between European pig breeds using microsatellite markers. Animal Genetics, 37:189–198.

Shriver MD, Jin L, Boerwinkle E, Deka R, Ferrel RE, Chakraborty R. 1995. A novel measure of genetic distance for highly polymorphic tandem repeat loci. Mol Biol Evol, 12:914–920.

Sneath PHA, Sokal RR. 1973. Numerical Taxonomy. Freeman, San Francisco.

Takagi M, Okamura T, Chow S, Taniguchi N. 2001. Preliminary study of albacore (*Thunnus alalunga*) stock differentiation inferred from microsatellite DNA analysis. Fishery Bulletin, 99:697–701.

Van Oosterhout C, Hutchinson WF, Wills DPM, Shipley P. 2004. MICRO-CHECKER: software for identifying and correcting genotyping errors in microsatellite data. Molecular Ecology Notes, 4:535-538.

Wittke-Thompson JK, Pluzhnikov A, Cox NJ. 2005. Rational inferences about departures from Hardy-Weinberg equilibrium. The American Journal of Human Genetics, 76(6):967–986.

Wright S. 1969. Evolution and the Genetics of Populations, vol. 2: The Theory of Gene Frequencies. University of Chicago.
